# Parsimony, Exhaustivity and Balanced Detection in Neocortex

**DOI:** 10.1371/journal.pcbi.1004623

**Published:** 2015-11-20

**Authors:** Alberto Romagnoni, Jérôme Ribot, Daniel Bennequin, Jonathan Touboul

**Affiliations:** 1 Mathematical Neuroscience Team, CIRB—Collège de France (CNRS UMR 7241, INSERM U1050, Labex MEMOLIFE), PSL, Paris, France; 2 Group for Neural Theory, Laboratoire des Neurosciences Cognitives, INSERM Unité 960, Département d’Études Cognitives, École Normale Supérieure, PSL, Paris, France; 3 Géométrie et dynamique, Université Paris Diderot (Paris VII), Paris, France; 4 INRIA Mycenae Team, Paris-Rocquencourt, France; Hamburg University, GERMANY

## Abstract

The layout of sensory brain areas is thought to subtend perception. The principles shaping these architectures and their role in information processing are still poorly understood. We investigate mathematically and computationally the representation of orientation and spatial frequency in cat primary visual cortex. We prove that two natural principles, local exhaustivity and parsimony of representation, would constrain the orientation and spatial frequency maps to display a very specific pinwheel-dipole singularity. This is particularly interesting since recent experimental evidences show a dipolar structures of the spatial frequency map co-localized with pinwheels in cat. These structures have important properties on information processing capabilities. In particular, we show using a computational model of visual information processing that this architecture allows a trade-off in the local detection of orientation and spatial frequency, but this property occurs for spatial frequency selectivity sharper than reported in the literature. We validated this sharpening on high-resolution optical imaging experimental data. These results shed new light on the principles at play in the emergence of functional architecture of cortical maps, as well as their potential role in processing information.

## Author Summary

Brain areas receiving sensory input often show specific functional organizations whose layout may subtend perception. We aim at understanding possible principles shaping these architectures and their role in information processing. Cat primary visual cortex provides our choice model. Pinwheel singularities of the celebrated orientation map are characterized by a locally exhaustive and parsimonious representation. We mathematically establish that these principles would constrain the spatial frequency map to have dipolar singularities co-localized with the pinwheels. Computational simulations show that this architecture could allow trade-off in detecting orientation and spatial frequency, but for sharper selectivities than reported in the literature. We show on physiological data that a sharp drop indeed appears, consistently with balanced detection paradigm. Moreover, recent experimental data have evidenced the presence of pinwheel-dipole topology in cat visual cortex.

## Introduction

In the neocortex, the part of the mammalian brain in charge of higher functions, multiple sensory modalities are represented. Characterizing finely these functional and anatomical organizations has been a great success of the past decades, in part thanks to great advances in cortical imaging techniques, and we now dispose of a relative clear description of the neocortex architecture. However, the principles that govern these architectures, as well as their role in efficiently encoding and decoding information, remain largely unknown, and are central concepts for comprehending how the brain perceives and processes information [1].

The early visual cortex of higher mammals provides a particularly interesting framework since it contains the concurrent representation of multiple attributes of the visual scene, processed into parallel cortical maps whose layouts are commonly thought to be mutually dependent. Groups of neurons in this area are preferentially selective to one specific value for each attribute. For instance, in response to a drifting grating, neurons in the early visual cortex encode the orientation (OR) [2] of the stimulus as well as its spatial frequency (SF) [3]. The two-dimensional OR map is continuous and consists of regular domains where preferred OR varies smoothly together with singularities, the pinwheel centers (PC), around which all ORs are represented [4, 5]. Moreover, other features like ocular dominance [6] and the local nature of the visual scene are retinotopically encoded in the primary visual area [2]: the information of a specific zone of the visual scene is processed by nearby neurons [7], and brain areas organizing these neurons reproduce the same characteristics at several places into a quasi-periodic structure [8].

Within a fundamental domain around a PC, the OR map is locally exhaustive (all attributes are represented), yet it is parsimonious in the sense that any OR is represented along a single level set. These two principles constitute very natural candidates for organizing the maps, yielding specific zones receiving all the information of the visual scene in an economic manner. The study of the representation of other attributes may allow investigating whether these principles also constrain their layout.

Among other possible functional organization in the visual cortex, the SF has recently attracted much interest. A common view is that its organization is constrained to that of the OR in order to ensure a uniform coverage [9], i.e. an even representation of the pairs (OR,SF). This theory was supported by data reporting an orthogonal relationship between iso-SF and iso-OR lines [10, 11] or the fact that PCs shall be situated near extrema of the SF representation [12–15]. These evidences did not appear clearly across different species: while strong orthogonality has been reported at global scale in monkey [10], only a weak tendency to orthogonality was shown in ferret [11]. In cat, it remains a disputed issue. Indeed, it was recently shown that the distribution of angles between iso-OR and iso-SF lines were not peaked around 90 degrees: these are globally uniform, with a small bias towards alignment in the vicinity of PCs [16].

**Fig 1:**
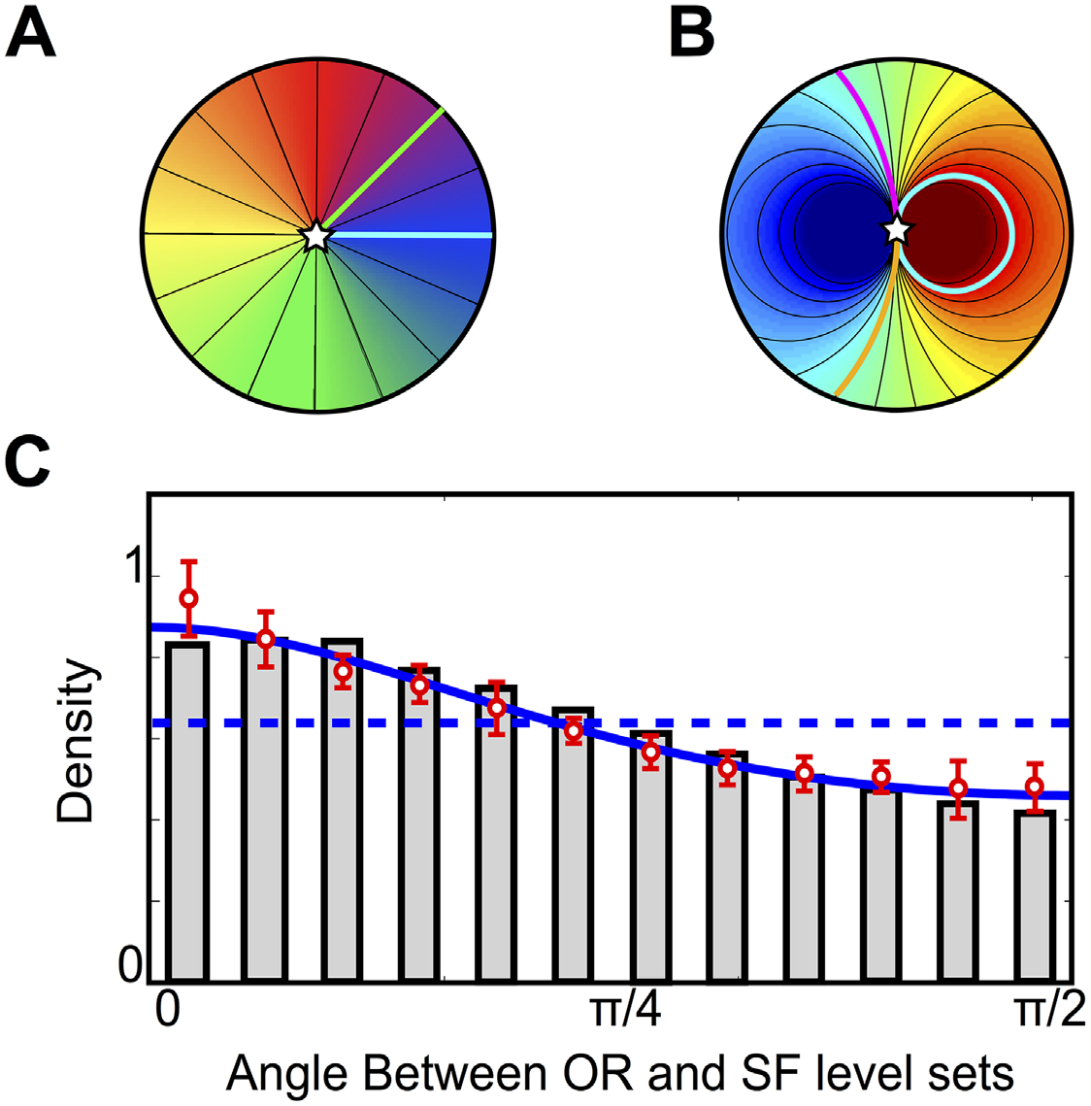
The pinwheel and dipole architectures and the intersection angles distribution. Two arbitrary level sets are represented: in the pinwheel topology (A), they connect the singularity (star) to the boundary, in the dipolar topology (B), they are either a single arc connecting the singularity to itself or made of two arcs connecting the singularity to the boundary. (C): Probability density of the angle between iso-OR and iso-SF lines. Red: experimental values of the mean (circles) and standard deviation (error bars) across the different cats (*n* = 4), within 150 *μm* from the PCs. Blue dashed line: uniform distribution for the dipole with SF map *γ*, gray bars correspond to the angle distribution of *γ* thresholded at ±1.4. Blue solid line: best fit with the analytically solvable model *γ*_*α*_ (here, *α* = 0.73).

This context motivated us to come back to this problem. We mathematically demonstrate here that SF representations that satisfy our two candidate principles, namely that are locally exhaustive and optimally parsimonious, organize around singular points into a universal topology evocative of an electric dipole potential (see Fig 1). This theory is particularly interesting since recent high resolution optical imaging data in cat [17] provide first evidences in favor of the presence of a continuous structure of SF maps with dipolar singularities co-localized with the PCs. Beyond the possible principles at the origin of this architecture, such organizations have important consequences on the coding capabilities of associated cortical areas. We show using a computational model that pinwheel-dipole (PD) architectures, even if they do not allow even representation of (OR,SF), may improve perceptual precision compared to the orthogonal architecture. Going deeper into the dependence of coding capabilities of PD architectures to SF selectivity, we realize that these organizations leave room for balanced detection of both attributes, but this occurs for SF selectivities sharper than the value previously reported in the literature [16]. Using finer estimates of the selectivity in the vicinity of PCs, we show indeed a clear sharpening of the SF selectivity near PCs perfectly consistent with the computational value predicted by the trade-off, leading to the natural prediction that PCs are singular locations of several maps at which selectivity ensures balanced detection.

## Results

### Universal topologies of minimal redundancy maps

Finding an optimal topology satisfying few simple conditions is easier said than done. A striking feature of the OR map is its very specific organization around singularities, the pinwheel topology, where the map is locally exhaustive and parsimonious. We will show that these two principles can characterize univocally the topology of maps representing periodic (e.g, OR) or non-periodic quantities (e.g, SF).

Because of the quasi-periodic structure of visual representations and the local nature of our criteria, we restrict our analysis to a small region of the visual cortex defined by an open set Ω, which is assumed to be, without loss of generality, a disc. The OR map is therefore defined as a continuous function $f:\Omega \;\mapsto \;S^{1}$ where $S^{1}$ is the circle [0, *π*] where we identify 0 and *π*. The SF map *g* is also defined on Ω, and takes values on an open (non-periodic) interval U⊂R. Maps will be said *exhaustive* on $S^{1}$ or *U* if their range covers the whole set of possible values.

The *topological redundancy* of a map is mathematically defined as the maximal number of connected components of the level sets. *Parsimonious maps* are those achieving the minimal redundancy possible. For instance, the *pinwheel topology*, corresponding to maps in which level sets are single arcs connecting a singular point to the boundary of Ω (see for example the case of the isotropic pinwheel represented in Fig 1A), has redundancy one and is hence parsimonious. Dipolar topologies correspond to maps whose level sets are made of two lobes of closed loops connecting the boundary to itself, completed by pairs of arcs connecting the singularity to the boundary of the domain. These have thus redundancy two, as is the case for example of the real-valued map plotted in Fig 1B. These two topologies are particularly important: indeed, we shall demonstrate that these are the unique topologies that are surjective (i.e., exhaustive) and minimize the topological redundancy (parsimony). We note that such maps necessarily show singularities. We concentrate on maps that are everywhere continuous except at isolated points. Such maps are referred to as *smooth simple maps*. We demonstrate in S1 Text section I the following:

**Theorem 1.** *Smooth simple maps that are exhaustive and parsimonious enjoy the following universality:*

1. *Smooth simple maps* $f:\Omega \;\mapsto \;S^{1}$ *that are exhaustive and with redundancy 1 have the topology of the pinwheel.*
2. *Smooth simple maps g*: Ω ↦ *U that are exhaustive and optimally parsimonious (redundancy 2) at arbitrarily small scales have the topology of the dipole.*

*Consequently, pairs of smooth simple maps* $\left(f,g\right):\Omega \;\mapsto \;S^{1}\times U$ *satisfying both exhaustivity and parsimony of each coordinate at arbitrarily small scales are the PD topology with co-localized singularities.*

This theoretical result is very general: it shows a universal property of maps satisfying local exhaustivity and parsimony principles. In particular, in view of our biological problem, shall the OR and SF maps satisfy these two principles, one will necessarily find PD structures in the vicinity of the PCs of the OR map. The proof of the theorem is based on (i) proving that the two principles impose that the maps have a singularity, and (ii) that the assumptions of the theorem constrain level sets to have precisely the desired topology.

This mathematical result has several implications that account for some experimental facts [16] inconsistent with the orthogonal architectures, including (i) the sharp transition of the SF map at PC locations, and (ii) the non-orthogonal distribution of angles between iso-OR and -SF lines.

Indeed, PD structures show a globally uniform distribution, with generically a small bias towards alignment for saturating models. In order to show the latter property, we shall study a simple model of PD architecture, with an SF map chosen in analogy with the electric dipole potential in 2 dimensions. In detail, the PD model is given by the dimensionless maps^1^ *φ*: *z* ↦ arg(*z*)/2 and γ:z↦Re(1/z) or, in polar coordinates for *z* = *re* ^*iϕ*^, $\varphi :z\;\mapsto \;\frac{\phi }{2}$ and $\gamma :z\;\mapsto \;\frac{\cos(\phi )}{r}$, respectively for the OR and the SF (see S1 Text section II for more details).

For this specific pair of maps, it is easy to show by direct calculation that the angle distribution is uniform. Although qualitatively consistent with the overall flat distribution reported in [16], it does not account for the slight over- (under)-expression of parallel (orthogonal) lines. This is due to the unrealistic sharp divergence of the SF representation of the electric dipole. Biological dipoles shall saturate to a maximal and minimal value at the singularity, and this saturation recovers this bias, as we show in the S1 Text. For instance, a simple generalization of the *γ*-map that allows for analytical developments, $\gamma_{\alpha }:\left(re^{i\phi }\;\mapsto \;\frac{\cos(\phi )}{r^{\alpha }}\right)$ with *α* < 1, which is less sharp than the *γ* map (but still diverging), reproduces the distribution of angles very accurately as we show in Fig 1C. The thresholded *γ* map also has the generic property of fitting accurately the distribution (Fig 1C), as generically do maps with SF saturating at the singularity.

These properties of PD architectures tend to point towards the fact that dipoles are consistent with previously reported facts on the behavior of the SF map at PCs. And recent optical imaging data have provided direct evidences of the presence of PD architectures using new high resolution optical imaging data to resolve the fine structure of the SF map on cat’s early visual cortex in the vicinity of PCs [17].

### Balanced detection of multiple attributes

**Fig 2:**
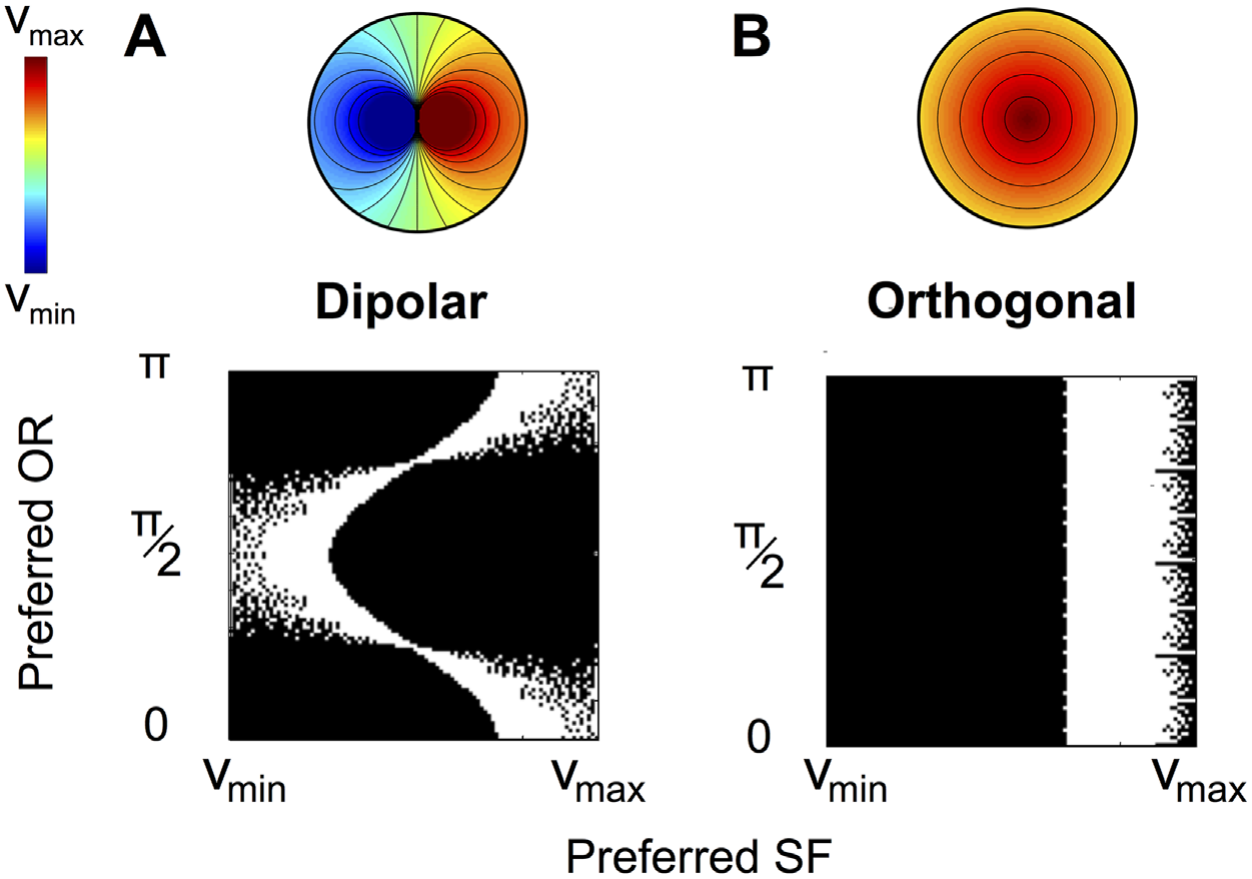
Parameter space coverage near PCs for dipolar and orthogonal architectures. Pairs of preferred OR and SF (white pixels) represented (A) in the dipole model and (B) in the putative orthogonal architecture. Corresponding SF maps displayed on top.

From the functional viewpoint, the fact that the PD architecture is highly non-orthogonal implies that the sampling of the attributes is not uniform near PCs. One may therefore expect the coding properties of the PD architectures to be very different than in a uniform coverage architecture. Under the uniform coverage assumption, orthogonality of level sets implies that the SF representation reaches a maximum (or a minimum) at the PC and smoothly decays away from the PC. Therefore, in a neighborhood of the PC, only a small portion of the range of SF is represented, contrasting with the PD architecture which is exhaustive. In particular, full representation of both high and low SFs in the orthogonal architecture would necessitate at least two PCs, one corresponding to a maximum of SF representation and the other one a minimum. At one given PC with orthogonal topology (say, corresponding to a maximum of the SF representation), it is likely that stimuli with high SF will be well encoded regardless of their OR, but may be blind to low SF stimuli. In contrast, the PD structure represents both high and low SFs, but different SFs are associated to distinct ranges of OR (see Fig 2). Overall, in both architectures, we find cells with preferred OR and SF covering the same proportion of possible stimuli, but with a different structure in the parameter space. It is therefore *a priori* unclear whether one of the architectures could present an advantage in perception capacity. In order to investigate this question, we simulated the activation of a piece of cortex with a PD or orthogonal architecture both fitted to the optical imaging data. We thus modeled the response of a cortical area to a given stimulus, and compared (i) the capacity of the PD and orthogonal architectures in discriminating two stimuli and (ii) the precision of the coding of both architectures. Models and results are described below, and more details on the data and models can be found in the Material and Methods section and S1 Text Section III.

#### Activity maps models

The PD model was directly fitted to the SF map (in logarithmic units—octaves) obtained by optical imaging data at around 200 PC locations (4 cats, 103 PC in A17 and 83 in A18) [17]. The orthogonal structure is not observed in our experimental data but was reported in previous studies [14]. The distribution of distances between PCs and the interval between minimal and maximal SF represented provide a natural slope of variation of an orthogonal model with linear SF radial variations. Both fits also provide the variability of parameters of each topology. The response curves (tuning curves) are peaked at the preferred OR and SF of the pixel, have a bell-shape classically modeled as (wrapped) Gaussian and thus only depend on a single parameter, the tuning width, or Full-Width at Half-Height (FWHH). The tuning widths were set to values that were previously reported in the literature. The OR tuning width was finely evaluated near PCs: *y*_*exp*_ = 80° [18]. Previous works reported only the value of the SF tuning width on the whole map (and not specifically near PCs); we thus initially used this value for our simulations: $w_{exp}^{all}=2.48$ octaves [16].

**Fig 3:**
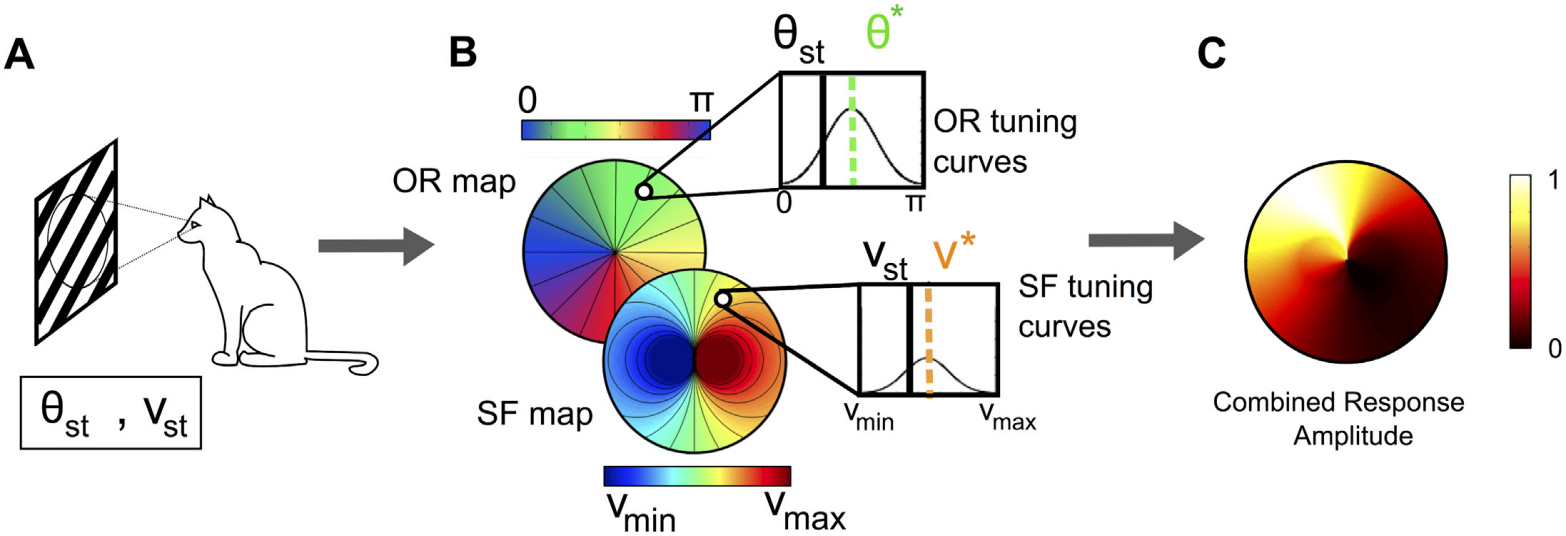
Activity maps generated from a given topology. (A): A grating with OR and SF (*θ*_*st*_, *ν*_*st*_) activates cells of the PA whose organization (preferred values (θ*, ν*)) is either assumed orthogonal or PD (B). The activity map, given by the level of activity of the different cells (C) is constructed using the tuning curves of the OR and SF.

Given a topology and provided that the response of each pixel to the stimuli is known, we can then compute the level of activity at each pixel, yielding an *activity map* (see Fig 3).

#### Discriminating between different stimuli

Based on these activity maps, we investigated the capability of PD and orthogonal architectures to discriminate between different stimuli. It seems reasonable to assume that the more different the activation maps the more efficient the discrimination, and this motivates to define the *discrimination level* between two stimuli (*θ*_*st*_, *ν*_*st*_) and (*θ*_*st*_ + *δθ*, *ν*_*st*_ + *δν*) as the $L^{2}$ distance between the two activation maps ∥Δ*A*∥_2_.

**Fig 4:**
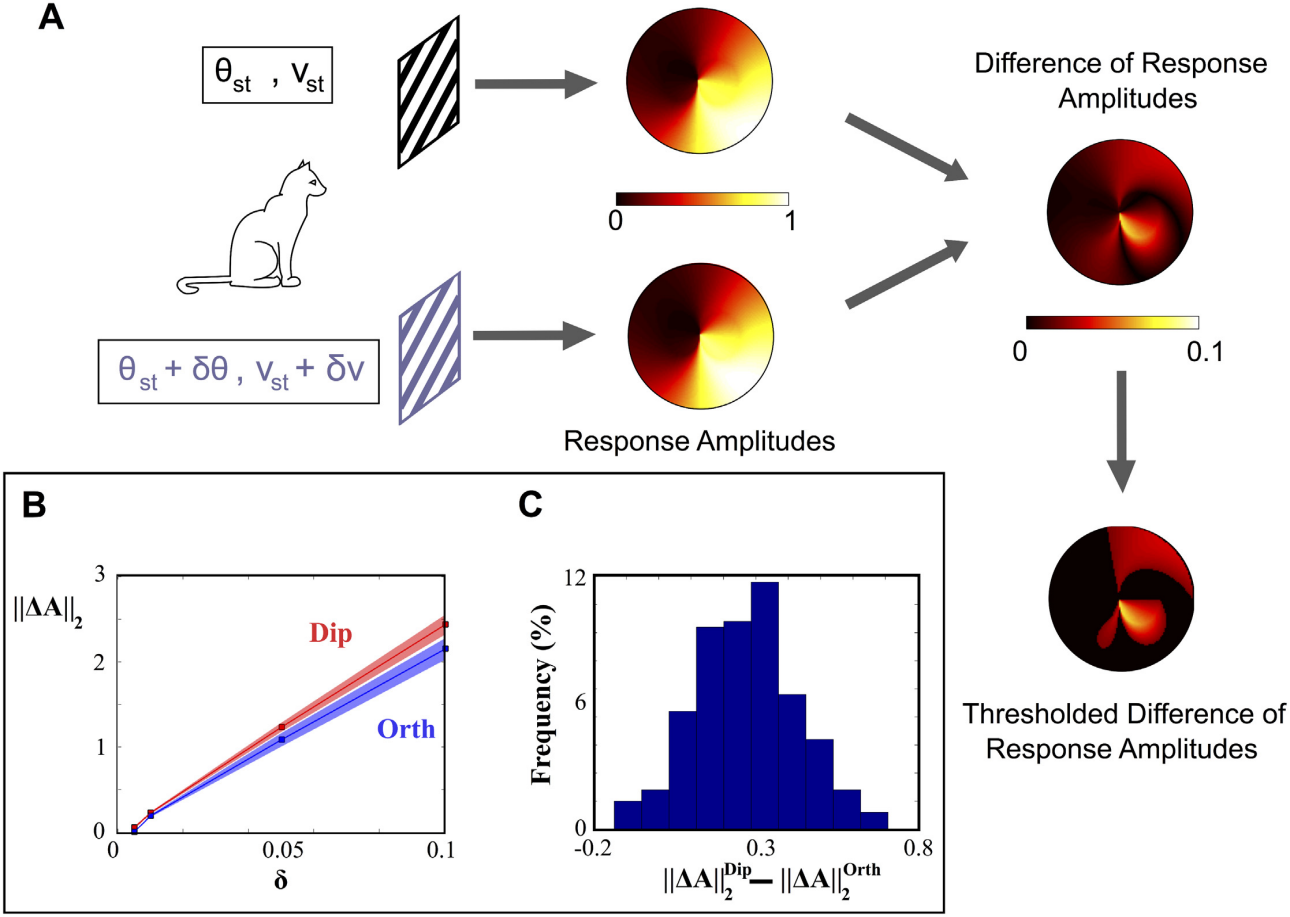
Discrimination levels of PD and orthogonal architectures. (A): Given two stimuli, activity maps are computed. The discrimination level ∥Δ*A*∥_2_ is given by the norm of the difference between the two maps (pixels with a difference smaller than 1% are not considered in the evaluation of the norm). (B) Median value (± mad) of ∥Δ*A*∥_2_ as a function of the distance *δ* between the presented stimuli in the parameter space $\delta =\sqrt{{\left(\delta \theta /\pi \right)}^{2}+{\left(\delta \nu /\Delta \nu_{extr}\right)}^{2}}$ for the dipolar (red) and orthogonal (blue) architectures. (C) Distribution of the difference between the median values (taken over stimuli) of the discrimination levels of dipolar and orthogonal architectures.

We computed the median discrimination levels across 100 random stimuli uniformly chosen in the range of possible stimuli experimentally used, for a choice of *N*_*PC*_ = 200 PD and orthogonal architectures drawn within the range of variation of the fits of the models on the data. The results (see Fig 4) indicate that dipolar architectures show larger discrimination levels than orthogonal architectures at all distances *δ* between the presented stimuli. In particular, we have found that only 5% of the tests provided a better discrimination to the orthogonal architecture, and these only occur when comparing the responses of very similar stimuli. In order to establish quantitatively this fact, we have computed the mean (± std) values for the difference between the two architectures and found that it is ${∥\Delta A∥}_{2}^{\text{Dip}}-{∥\Delta A∥}_{2}^{\text{Orth}}=0.27\pm 0.16$. This indicates that the discrimination level of the PD architecture is significantly larger than that of the orthogonal architecture (*Z*-score = 1.69, one-tailed *p*-value *p* = 0.045). This finding indicates that in a discrimination task between two stimuli, the PD topology could locally present an advantage over the orthogonal topology.

#### Decoding, balanced detection and the selectivity sharpening

**Fig 5:**
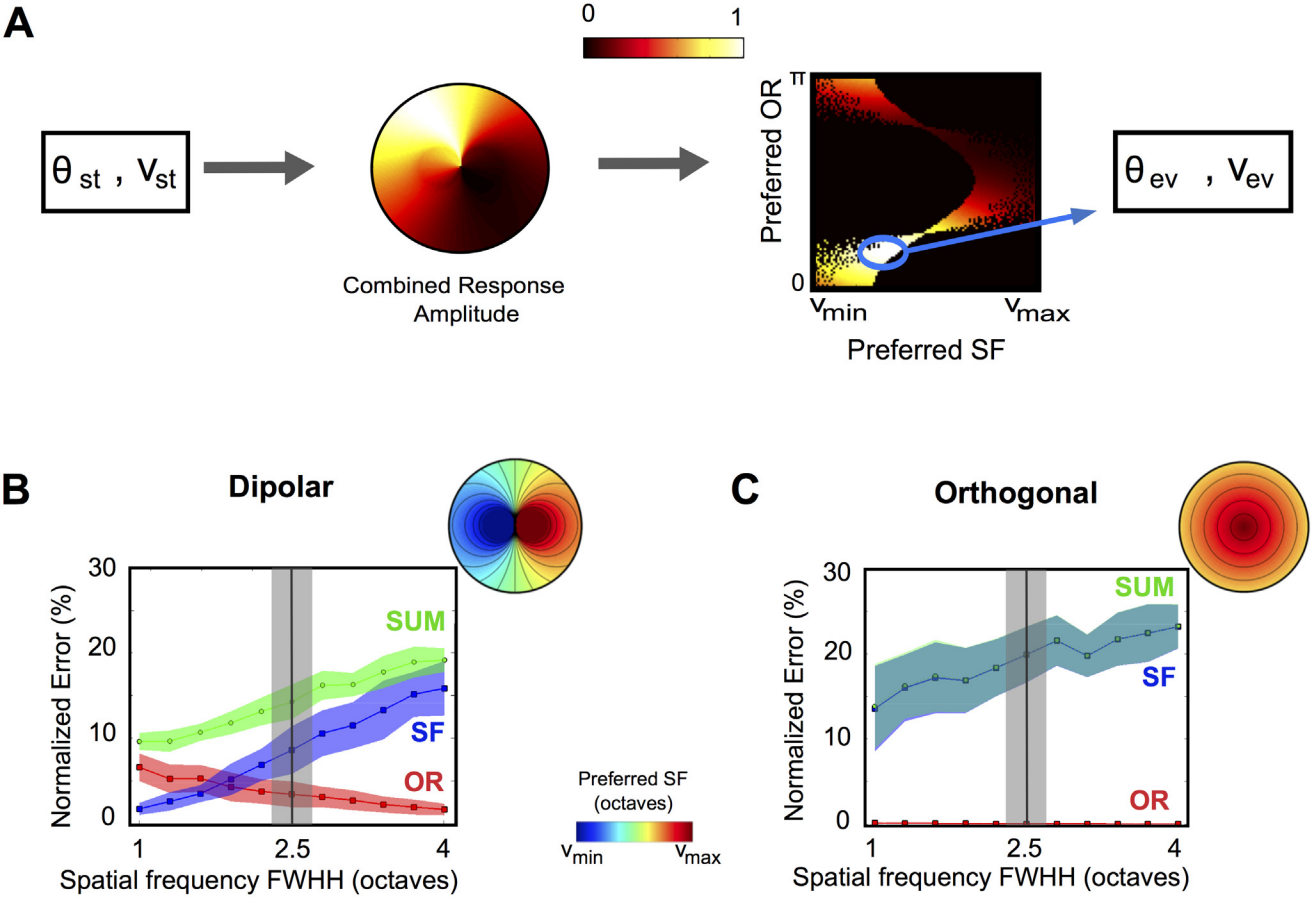
Decoding an OR and SF stimulus, and balanced detection at PCs. (A): Decoding algorithm: the activity map in response to a stimulus (*θ*_*st*_, *ν*_*st*_) is represented in the parameter space of preferred (OR, SF). An estimated stimulus (*θ*_*ev*_, *ν*_*ev*_) is obtained by the weighted average of the 10% highest response amplitudes. (B): Median error (in %± mad) in the detection of OR (red), SF (blue) and OR + SF (green), for the dipolar structure as a function of the FWHH for the SF tuning curve (in octaves). The FWHH for the OR tuning curve is set at *y*_*exp*_ = 80°. The gray bar corresponds to the experimental variability of the SF tuning [16] of the whole map. One example of the SF maps considered is shown on the top-right corner. (C): The same as (B) for the orthogonal architecture.

The activity maps computed above are the neural correlates of the presence of a stimulus. From this pattern of activity, the brain extracts information on the visual scene, using a procedure which is largely unknown. In order to characterize more finely the coding efficiency of both architectures, we developed a simple procedure to decode the stimulus by estimating it from an activity map and given the pair of preferred OR and SF maps. The decoding algorithm consists in evaluating the stimulus as the center of mass of the responses in the preferred (OR,SF) plane (Fig 5A). The estimation errors (*ϵ*_*θ*_, *ϵ*_*ν*_) are defined as the difference between the actual and estimated stimuli normalized by the range of represented OR and SF; a combined error can be formally defined as *ϵ*_*tot*_ = *ϵ*_*θ*_ + *ϵ*_*ν*_. We have compared the distribution of errors (over 100 randomly chosen stimuli per architecture) computed on *N*_*PC*_ = 50 PD and orthogonal topologies drawn within the range of variation of the experimental data.

The simulation results indicate that the accuracy of OR detection in the dipolar structure is significantly degraded compared to the orthogonal one *ϵ*_*θ*_ = 3.4 ± 1.2% vs 0.06 ± 0.01% (median normalized error ± mad; Mann-Whitney-Wilcoxon test, *p* < 10^−3^). Nevertheless, the SF detection is vastly improved, *ϵ*_*ν*_ = 8.1 ± 1.8% vs. 20 ± 3% (*p* < 10^−3^). We note that while the error made in detecting ORs in the dipolar model remains reasonable, the error in detecting SF at a single pinwheel by the orthogonal model is very large. This is related to the fact that a single pinwheel in the orthogonal architecture represents only a small portion of the SF range.

These performances in evaluating the correct stimuli are expected to vary depending on the SF tuning width, since this parameter strongly constrains the responses of the cortical area and particularly the coverage of the feature space (OR, SF) as depicted in supplementary S4 Fig. Indeed, enlarging SF tuning width improves the detection of OR since the coverage of the induced activity in the feature space (OR, SF) increases (Fig. S4 in the S1 Text), and degrades fine detection of SF. In order to investigate the importance of this dependence on selectivity, we therefore simulated the model for distinct values of the SF tuning width (Fig 5), both for the PD and for the orthogonal architecture. Depending on the architecture, we observed very distinct qualitative behaviors of the errors when the SF tuning width is varied. As expected, the error in perceived SF (blue curve) increases with the SF tuning width in both architectures. In the orthogonal architecture (Fig 5B), the error in perceived SF remains much higher than the one in perceived OR (Mann-Whitney-Wilcoxon test, *p* < 10^−3^).

In the dipolar architecture, as the tuning width decreases, the error in perceive OR goes from very small to very high values, opposite to the variation of the error in detecting SF. Therefore, the SF and OR error curves necessarily intersect. This indicates that there exists a specific tuning width for which our estimates of the error in detecting OR and SF are equal. We say that the system shows at this point a *balanced detection* of the two attributes; because of the opposite monotonicity of the SF and OR error curves, balanced detection requires a trade-off between the levels of errors of the two attributes. The tuning width corresponding to the intersection of the two curves is estimated to be $\bar{w}=1.79\pm 0.25$ octaves, which is significantly below $w_{exp}^{all}$ (*Z*-score = 1.83, one-tailed *p*-value *p* = 0.03).

**Fig 6:**
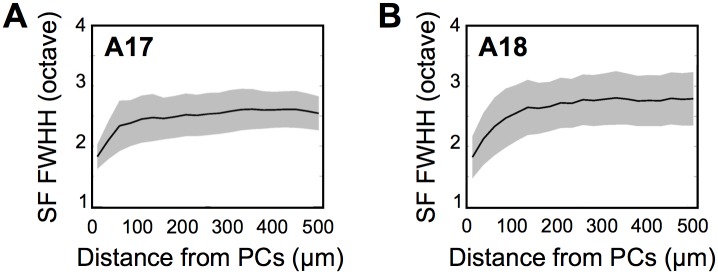
SF tuning width drops close from PCs. (A): Median FWHH_SF_ (black curve) ± mad (gray) as a function of the distance from PCs in A17. Each point corresponds to a distance of 25 *μ*m. (B): The same as (A) for A18.

In order for the system ensure balanced detection of both attributes at PC, the SF tuning curve shall sharpen in the vicinity of the singularity. To assess whether the system is indeed poised at this particular point, we came back to our experimental data and investigated finely the SF tuning width and its dependence as a function of the distance to the set of PCs. Strikingly, our data showed that SF tuning width sharply drops close to PCs (at a distance of around 100 *μm*, see Fig 6), reaching the value (median ± mad) $w_{exp}^{PC17}=1.83\pm 0.20$ octaves in A17 and $w_{exp}^{PC18}=1.83\pm 0.24$ octaves in A18, within 25 *μm* from PCs. The sharpening is clearly visible in the raw response curves found in optical imaging as we present in S5 Fig. These measurements are therefore consistent with the point of balanced detection determined theoretically (A17: *Z*-score = 0.11 two-tailed *p* = 0.91; A18: *Z*-score = 0.09, *p* = 0.93).

We emphasize that this level of SF tuning width is in particular neither optimal for the SF detection alone (occurring for relatively small SF FWHH), nor for the detection of OR alone (corresponding to large SF FWHH), and nor for the combined error of OR and SF detection (green curve), that linearly increases with *w*. Therefore this argues that selectivity properties near PCs favor a trade-off in the detection precision (as we quantified through normalized errors) of the two attributes.

One may wonder whether the advantage of the dipolar architecture over the orthogonal architecture in the discrimination task persists for this SF FWHH. We thus computed the discrimination levels obtained with SF tuning width fixed at $w_{exp}^{PC17}$. We confirmed that, although less significantly than at $w_{exp}^{all}$, this sharper selectivity does not modify the fact that the PD topology again shows better discrimination than the orthogonal one (mean difference ${∥\Delta A∥}_{2}^{\text{Dip}}-{∥\Delta A∥}_{2}^{\text{Orth}}=0.24\pm 0.24$, *Z*-score = 1, one-tailed *p*-value *p* = 0.15).

#### Robustness of the results for the trade-off

In this analysis, we used a model of dipole which, while fitted with accuracy to the optical imaging data, imposes a specific radial profile of the SF map. One may therefore wonder whether the emergence of a balanced detection between OR and SF qualitatively and quantitatively persists across different dipolar architectures, i.e. to what extent the balanced detection occurring in our dipole model is robust to modifications of the model of dipole chosen. One natural generalization of the model that we already introduce in this manuscript consists in considering dipoles with distinct SF map profiles given by the class *γ*_*α*_. All members of this class conserve the dipolar topology but have different shapes (e.g. different distributions of iso-OR and iso-SF angles, see Fig 1C) and may be related to different error curves.

**Fig 7:**
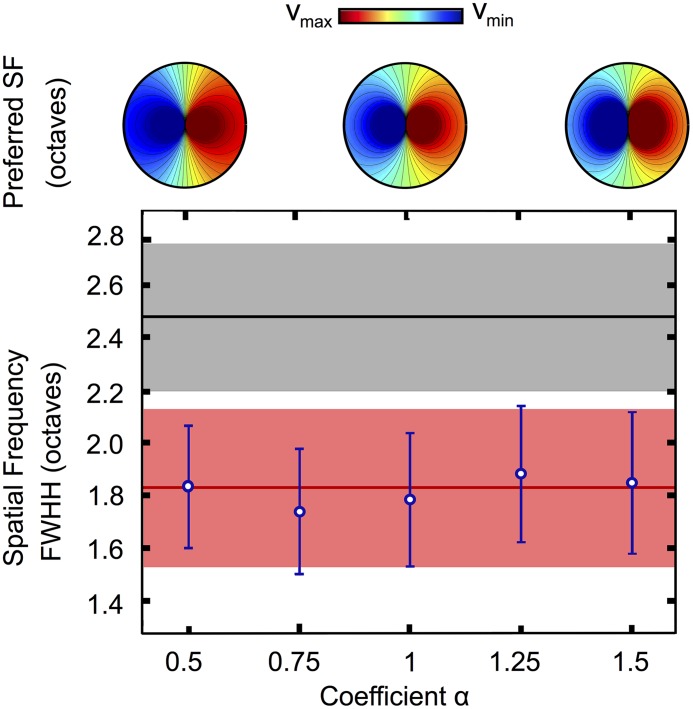
Robustness of the balanced detection for dipolar architectures *γ*_*α*_. Top: fitted architectures for *α* = 0.5, 1, 1.5 visually look very different. Bottom: SF tuning corresponding to balanced detection as a function of *α*, computed with the same procedure as in Fig 5. The gray band corresponds to the SF tuning width on the whole map $w_{exp}^{all}$ and the pink band to our estimate in the vicinity of PCs $w_{exp}^{PC17}$ (Fig 6). Blue circles and error bars correspond to the mean and standard deviation of the SF FWHH at the tradeoff for different values of the parameter *α*.

We again fitted the all models to the data, allowing for angular and asymmetry deformations of this model for different values of *α*. The thus fitted average dipoles visually look relatively different (see Fig 7). These different best fitted architectures were used to test the error made on the OR and SF evaluation. In all cases, curves of errors in OR and SF keep the same monotonicity as the SF tuning width was varied, and intersect. The values of the intersection were computed (with *N*_*PC*_ = 200) and reported in Fig 7, bottom panel. Independently on the parameter *α*, they are not consistent with $w_{exp}^{all}$ (*Z*-score ≥ 1.55, one-tailed *p*-value ≤ 0.06), but actually always compatible with $w_{exp}^{PC17}$ (*Z*-score ≤ 0.24, two-tailed *p*-value ≥ 0.81).

This finding points towards the fact that (i) the topology and (ii) the particular scales and shapes found in the experimental data are central to fix a value of the tuning width for the balanced detection $\bar{w}$, probably more than the details of the model. These results therefore tend to stress that not only the presence of a trade-off between the two normalized errors is a natural feature of the PD architecture, but also that, regardless of the precise profile of the theoretical map, this fact takes place for SF selectivities in perfect agreement with the biological measurement of SF selectivity near PCs.

## Discussion

This study showed that optimizing simple criteria strongly constrains the layout of maps, and that these layouts can provide specific coding capabilities. We concentrated here on two principles, local exhaustivity and parsimony, which imply that both maps shall display co-localized singularities, around which the OR map is organized as a pinwheel and the SF map as a dipole. While pinwheels were identified since decades [4], dipoles were never observed in previous studies. In a companion paper [17], high resolution optical imaging made it possible to observe these topologies and validate quantitatively the presence of PD singularities. It is striking that both maps satisfy the same optimality properties near the same singularities. At the scale of the whole map, fine detection of local characters in the visual scene makes optimal parsimony not desirable: it is rather important to be able to have accurate detections at several places of the visual scene. Principles at play in the architecture of the whole map shall therefore take into account this necessity, as well as some invariance principles [8, 19] that may fix the density of singularities with respect to a map typical scale. Moreover, purely global criteria such as continuity and coverage are often not sufficient to reproduce quantitatively the architectures of maps in the early visual cortex [20–22]. It is likely that its overall structure emerges from a compromise between local criteria, invariance principles and global continuity-coverage optimization.

Locally around the singularity, we showed that the maps organization has important implications in coding capabilities. It is noticeable that the PD architecture allows for a balanced detection of the OR and the SF, but for tuning widths smaller than the value reported in the literature. This fact did not depend tightly on the choice of the model: it was consistently obtained qualitatively and quantitatively over a class of functions fitted to the experimental data. This has motivated us to estimate more finely the SF tuning width in the vicinity of pinwheels. Consistently with the balance detection point identified in the model, we observed a sharp decrease of the SF tuning width statistically compatible with the model’s prediction. This is a surprising phenomenon: the sharpening of the SF tuning curve contrasts with the well-documented broadening of OR selectivity near pinwheels [18, 23]. It is interesting to note that sharpening cannot be an artifact of the presence of a singularity, at which subsampling related to the imaging resolution may induce rather a broadening of the selectivity as in the OR case. This significant sharpening of the SF selectivity assembles with a number of other peculiar properties of cells in the vicinity of PCs, including their increased resistance to monocular deprivation [24], enhanced sensitivity to OR adaptation [25] and distorted retinotopic representation [26], pointing towards a very specific role of pinwheel location in the visual system.

It is worthwhile noting that psychophysics data [27, 28] in cat report estimates of the visual acuity in OR and SF consistent with balanced normalized errors. Moreover, the normalized error of OR and SF visual acuity of cat falls within the range of 1 − 5% which is precisely the level of normalized error at the intersection of OR and SF error curves. This agreement is very surprising given that our estimates postulate only a very simple decoding algorithm and do not take into account the coding of regions away from PCs. Processing of the visual information likely uses more information and may take advantage of both preferred attributes and regions of maximal sensitivity. From the information theory viewpoint, an important theoretical question is to characterize topologies maximizing information capacity for multiple attributes representation, in the vein of the studies on OR only [29]. Eventually, extending the analysis to the properties of multiple pinwheels (thus incorporating the properties of regular domains) is an important endeavor that requires a more detailed experimental characterization of the organization of maps away from PCs.

From a biological viewpoint, it would be worthwhile to investigate whether this study can be extended beyond the case of the OR and SF maps of the cat. This would necessitate to record functional maps for other attributes, and check whether these satisfy exhaustivity and parsimony of representation, if the singularities of OR-SF maps are special points of these maps, and if selectivity properties adjust to ensure perceptual trade-offs. It should not be surprising that other principles constrain the layout of other maps. In particular, the direction preference map is not exhaustive near pinwheels, and is discontinuous [30], because it is constrained to the OR map. Another example is given by the binocular disparity map [31], which may be optimized to ensure the even representation of visual input from both eyes [32] and by the ocular dominance map which was shown to be orthogonal to the OR [5] and whose role in perception remains to be completely understood [33].

From a mathematical modeling viewpoint, the good agreement between the theoretically derived model and new data constitute an encouraging step towards the development of more complex models that could account for higher order visual areas processing more complex features.

## Materials and Methods

The full mathematical framework is provided in the S1 Text. The computational developments are based on models of PD architectures and models of detection (coding and decoding the features of a visual stimulus), which were both fitted to optical imaging data. We present here in detail these models, as well as the data we used to fit the models and to estimate the sharpening of selectivity near PCs.

### Ethics statement

Experiments were conducted on 4 young adult cats aged between 24 and 72 weeks. Animals were anesthetized, paralyzed, and artificially ventilated with a 3:2 mixture of N2O and O2 containing 0.5–1% isuflurane. All experiments were performed in accordance with the relevant institutional and national guidelines and regulations (i.e., those of the Collège de France, the CNRS, and the DDPP). Experiments also conformed to the relevant regulatory standards recommended by the European Community Directive and the US National Institutes of Health Guidelines.

### Models of PD architectures and fits

In order to investigate the different efficiencies of the PD and orthogonal architectures, we have simulated circular regions Ω of radius *R* = 50 pixels. Each pixel of these discs represents a set of neurons responding to a specific range of OR and SF, distributed around the singularity at the origin. The amplitude of their response to varying external stimuli produces graded responses, peaked at a specific value (the quantity represented in the corresponding functional map) and well approximated by the product of a wrapped Gaussian function, for the OR coordinate, and a Gaussian function, for the SF one (the tuning curves, see Fig 3B) [34].

The OR map has been defined as half of the polar angle, modulo an arbitrary phase. For the dipolar SF map we studied the class of functions *γ*_*α*_, saturating at extreme values and incorporating possible angular and shape deformations. For the orthogonal case, we considered a rotational invariant map, linearly decreasing from a maximum value located at the origin.

The realistic range of values for the free parameters of the PD model have been evaluated by fitting to the optical imaging data (restricting to fits with coefficient of determination > 0.8, using Matlab function regress). The same data have been used to estimate the slope of the radial SF decay parameter in the orthogonal model. For both PD and orthogonal cases, we have simulated 50 different couples of OR and SF maps, differing each other for the parameter sets chosen in these intervals. Details of the models and functions used are provided in S1 Text section III (as well as in the papers [16, 17]).

### Optical imaging data

High-resolution intrinsic optical imaging was performed in cat visual cortical areas A17 and A18 to record maps of OR and SF. All experiments were performed in accordance with the relevant institutional and national guidelines and regulations (i.e., those of the Collège de France, the CNRS, and the DDPP). Experiments also conformed to the relevant regulatory standards recommended by the European Community Directive and the US National Institutes of Health Guidelines. A complete and in depth description of the experimental protocol is detailed elsewhere [16, 17].

#### Animal model and surgical procedure

Experiments were conducted on 4 young adult cats aged between 24 and 72 weeks. Animals were anesthetized, paralyzed, and artificially ventilated with a 3:2 mixture of N2O and O2 containing 0.5–1% isuflurane. Electrocardiogram, temperature, and expired CO2 were monitored throughout the experiment. Animals were installed in the Horsley-Clarke stereotactic frame and prepared for acute recordings. The scalp was incised in the sagittal plane, and a large craniotomy was performed overlying areas 17 and 18 of both hemispheres. The nictitating membranes were retracted and the pupils were dilated. Scleral lenses were placed to protect the cornea and focus the eyes on the tangent screen 28.5 cm distant.

#### Optical imaging

The cortex was illuminated at 700 nm to record the intrinsic signals. The focal plane was adjusted to 500 *μ*m below the cortical surface. The optic discs were plotted by tapetal reflection and the center of the screen was moved 8 cm (15°) below the middle of the two optic discs. Intrinsic optical signals were recorded while the animals were exposed to visual stimuli displayed on a CRT monitor subtending a visual angle of 75° × 56°. Frames were acquired by CCD video camera (1M60, DALSA) at the rate of 40 frames per second and were stored after binning by 2 × 2 pixels spatially and by 12 frames temporally using the LongDaq system (Optical Imaging). Images were acquired with a resolution of 15.3 or 5.9 *μ*m/pixel.

#### Stimulation

Each stimulus consisted of full-screen sine-wave gratings drifting in one direction and rotating in a counter-clockwise manner [35]. The angular speed of the rotation was 2 rotations per min and the temporal frequency of the drift was 2 Hz. The contrast was set at 50%. Thirty SFs ranging linearly in a logarithmic scale from 0.039 to 3.043 cycle/degree (cpd) were presented in random order. Ten full rotations were presented for each SF. At the end of the last rotation, the first frame of the first rotation for the next SF was presented without interruption. The total duration of the recording was 2.5 hours.

#### Image processing

Data were pre-processed with the generalized indicator function method [35] for each SF separately. A low-pass filter with a Gaussian kernel of around 15 *μ*m half width was also applied for smoothing the data. A Fourier transform was performed on the temporal signal of each pixel for all SFs together. The phase at half the frequency of rotation was calculated to obtain the preferred OR at each pixel [35]. Then intrinsic signals related to each SF were considered separately. For each pixel, the modulation of the signal induced by the rotation of the gratings was interpolated via a least-square method with a cosine function whose phase was equal to the preferred OR at this pixel and whose frequency was equal to half the frequency of rotation. Magnitude maps for preferred ORs were thus obtained for each stimulus SF [16]. Pixels with negative values, which corresponded to interpolation peaking at orthogonal ORs, were rectified to zero. Then, at each pixel, the intrinsic signals were interpolated with a difference of Gaussians function and three parameters were extracted: the preferred SF, the full-width at half-height and the error-of-fit.

## Supporting Information

S1 Text Details for mathematical proofs, models and numerical simulations. (PDF)

S1 Fig Topologies. The 8-shapes bouquet (A) and the topology of the dipole (B), with an isolated defect.
(TIFF)

S2 Fig Intersection angles distribution. Distribution of the angles between the level sets of orientation and spatial frequency maps locally around the pinwheels for different models. The black dots and the error bars represent respectively the means and standard deviations with respect to different cats from experimental data. Red crosses and blue circles corresponds respectively to the numerical results for the maps *γ*_*B*_ and *γ*_*C*_ defined in the S1 Text.
(TIFF)

S3 Fig Estimation of the parameters for the orthogonal architecture. (A): Schematic representation of the model used for the orthogonal architecture (adapted from [15]). In this model, iso-orientation lines (colored) intersect at pinwheel centers that tend to lie over low (red) and high (blue) SF domains. In order to represent all combinations of OR and SF equally, SF was assumed to linearly vary between the two pinwheels separated by a distance d. The value of d was defined as the median minimum distance (± mad) from one pinwheel to another whose histogram is shown in (B).
(TIFF)

S4 Fig Representation of the induced activity in the parameter space. Relative response of dipolar (A) and orthogonal (B) architectures near pinwheel centers to external stimuli in the parameter space. For any given pixel (θ*, ν*), we show the normalised sum of all the responses *F*_θ* ν*_(*θ*_*st*_, *ν*_*st*_) (Eq. S10, S11 and S12 in S1 Text, with parameters *σ*_*OR*_ = 0.63 and *σ*_*SF*_ = 1 octaves) with stimuli (*θ*_*st*_, *ν*_*st*_) spanning the same parameter space. The normalisation is chosen by taking the minimum and maximum values of the two combined distributions. The relative distributions are represented in the histograms in the top-right corners.
(TIFF)

S5 Fig SF tuning curves sharpen close to a PC. SF tuning curve relative to cortical area in the white circle in A are represented in B. Black curves represent pixels with a high error-of-fit (> 0.5). These are represented in the close vicinity of the PC. Gray curves represent pixels with an acceptable error-of-fit (< 0.5). Although most of these pixels are located further away (See C, D, F, G for instance), some are also located close to the PC (E). Surprisingly, the later pixels exhibit a sharp selectivity for SF preference.
(TIFF)

## Footnotes

^1^ Here, Ω is assumed to be the unit disc {|*z*|<1} of the complex plane and *z* = *re^iφ^* is the complex coordinate.
