# Supplementary material for: Parsimony, Exhaustivity and Balanced Detection in Neocortex
Source: PLoS Comput Biol. 2015 Nov 20;11(11):e1004623. doi: 10.1371/journal.pcbi.1004623 (PMC4654526; doi:10.1371/journal.pcbi.1004623)
Supplement: S1 Text — (PDF) [file pcbi.1004623.s001.pdf]

Parsimony, exhaustivity and balanced detection in neocortex: SUPPORTING INFORMATION

Alberto Romagnoni Jerôme Ribot Daniel Bennequin Jonathan Touboul

I Pinwheel-Dipole architecture minimizes geometric redundancy

We provide here the details of the mathematical analysis of the topology of local representations of OR and SF. We will be particularly interested in the parsimony and exhaustivity of the representations of these maps. We will show that the pinwheel architecture optimizes the geometric redundancy of representation of a periodic quantity (the orientation), while the dipole optimizes the geometric redundancy of representation of a non-cyclic variable (the spatial frequency). These results go beyond the example of OR and SF representations: a number of sensory modalities have a local character, e.g. touch or vision. Some of the neurons processing this local information have receptive fields that reflect the local domains of sensor cells. In general these receptive fields shall have access to other characteristics of the stimuli, and fine perception necessitates that brain areas reproduce the same characteristics many times, in several places: in our context, early visual cortex of the cat shows quasi-periodicity and continuity of the representation. We will establish that the minimization of redundancy in local domains (i.e. fundamental cells of the quasi-periodic maps) explains most of the experimentally observed structure.

In detail, we show in the case of the OR representation (corresponding to a continuous map with value in a circle) that minimal redundancy 1 is equivalent to the pinwheel architecture. As for SF, we prove that, to represent a real variable which varies in an open interval (or equivalently, from $-\infty$ to $+\infty$), several constraints arise: i) the map must have singularities (points where the value is undefined), and ii) near such singularities of the map, the dipolar architecture is the unique structure that is compatible with continuity, and has minimal redundancy.

It is remarkable that the pinwheel-dipole architecture observed in the cat's visual cortical areas 17 and 18 in the present work is the unique scale-invariant topology achieving minimal representation of a pair (θ, ν) for the the circular variable θ and the variable ν taking values in an open interval.

I.I Topological Redundancy, Pinwheel and Dipole Topologies

Since the OR and SF maps are quasi-periodic, we limit our study to a fundamental domain Ω, having the topology of a disc. Without loss of generality, we will hence consider that Ω is disc centered at the origin 0, and denote by Γ the boundary circle of Ω. Coordinates in this disc are denoted (x, y). The letter u denotes the scalar variable to be represented. In one dimension, there are only two types of smooth domains, the open interval U (also isomorphic to the real line $\mathbb{R}$), and the circle $\mathbb{S}^1$ (a closed periodic interval). A cortical map f is an application from Ω on $\mathbb{S}^1$ (e.g., the OR map) or U (e.g., the SF map). In $\mathbb{S}^1$ we shall generically choose a coordinate θ defined modulo π.

We now formally define pinwheel and dipole topologies, examples of which were represented in Fig. 1.

Definition I.1. *We consider a map $f : \Omega \mapsto \mathbb{S}^1$ and $g : \Omega \mapsto U$. We say that:*

1. *the f has the* pinwheel topology *if it has a singularity (say, without loss of generality, located at the origin 0) and each level set connects the singularity to the boundary Γ of Ω. Figure 1A represents a typical example*[1].

[1] Any map with the pinwheel topology is equivalent to that topology in the sense of homotopy, i.e., there exists a continuous and invertible map transforming the map into $\Omega : re^{i\phi} \mapsto \frac{\phi}{2}$.

2. *g has the* dipolar topology *if (i) it has a singularity, (ii) its level sets are either made of single closed arcs connecting the singularity to itself or two arcs connecting the singularity to the boundary, and (iii) it has exactly two disjoint families of loops connecting the singularity to itself. Fig. 1 B is a typical example of such topology.*

As noted in the main text, pinwheel topology has geometric redundancy 1, and is moreover scale invariant in the sense that both exhaustivity and redundancy 1 persist in any smaller neighborhood of the singularity (e.g., in Fig. 1A, for any smaller circle around the singularity). Similarly, dipoles have topological redundancy 2, and scale invariance property: all levels are represented arbitrarily close from the singularity and the geometric redundancy is two on any circle, arbitrarily small, around the singularity.

I.II Minimal representations of orientations and universality of the pinwheel

In this section, we show that pinwheel architecture is the unique minimizer of geometric redundancy allowing exhaustive representation of all ORs in Ω. The proof proceeds by showing that the representation cannot be minimal without presenting at least one singularity in Ω, and that all level sets connect that singularity to the boundary.

Theorem I.1. *A continuous surjective map $f : \Omega \mapsto \mathbb{S}^1$, with redundancy 1, has the topology of the pinwheel.*

Proof: Let $\theta_0 \in f(\Gamma)$ be a fixed value. The level set $f^{-1}(\theta_0)$ is a connected set C intersecting Γ. The complementary of C in Ω cannot have strictly more than two components: indeed, if that was the case, the continuity of f would imply that there exist at least two connected components of $f^{-1}(u)$ for a value of u near θ_0 contradicting the redundancy 1 assumption.

If the level set C splits Ω into two disconnected components: Ω_1 in which the values of f are larger than θ_0 in the neighborhood of C, and Ω_2 where f is locally smaller. We denote by $I_1 = [\theta_0, \theta_1]$ the interval of values covered on $\Omega_1 \cup f^{-1}(\theta_0)$ (considering a continuous in version of the argument, i.e. we have possibly $\theta_1 > \pi$) and $I_2 = [\theta_2, \theta_0]$ on $\Omega_2 \cup f^{-1}(\theta_0)$ (with, again, θ_2 possibly smaller than 0). Since both sets are compact, these values are reached. Then if $\theta_1 \geq \theta_2 + \pi$, the redundancy is greater than 2, and if not there is an interval of $\mathbb{S}^1$ that is not covered by f contradicting surjectivity.

The level set therefore does not splits Ω into disconnected components. There are two possibilities: either $C \neq \Gamma$, discarded using the same argument as above, or C enters the interior of Ω and necessarily ends at one point z^*. Let us fix two points z_1 and z_2 outside of the level set, and two arcs γ_1 and γ_2 connecting them, and such that γ_1 crosses C and γ_2 does not. The map f restricted to $\gamma_1 \cup \gamma_2$ therefore covers $\mathbb{S}^1$. This is true arbitrarily close to z^*, implying that z^* is a singularity and all level sets converge to it.

Proving that the map has the pinwheel topology only amounts showing that all levels are represented along the boundary. The map f restricted to Γ is continuous. If it does not cover $\mathbb{S}^1$ then along Γ, f takes necessarily twice the same value, contradicting redundancy 1 (since we have seen that any level set starting from the boundary reaches necessarily the singularity). Therefore, all levels are represented on the boundary Γ and reach the singularity: the map has the topology of the pinwheel. □

The topology of the pinwheel is therefore universal: any map minimizing redundancy of representation has this topology. An analogous property is now demonstrated for the representation of SF.

I.III Minimal representations of the spatial frequency and universality of the dipole

We examine now the topology of exhaustive and parsimonious continuous maps taking value in an open interval U. The logarithm of the SF is a non-periodic real quantity, and within the cortex, neurons respond to a specific range. This interval is not universal: it may vary across animals, and within the same animal across different regions. In that view, the representation of SFs cannot be considered within a closed interval. This justifies the choice of U as an open interval, which is mathematically equivalent to considering continuous representations of the whole real line. We will therefore consider in the rest of the section $U = \mathbb{R}$. Continuous maps defined on Ω with no singularity, cannot be surjective on $\mathbb{R}$. Indeed a continuous map on a compact set reaches finite

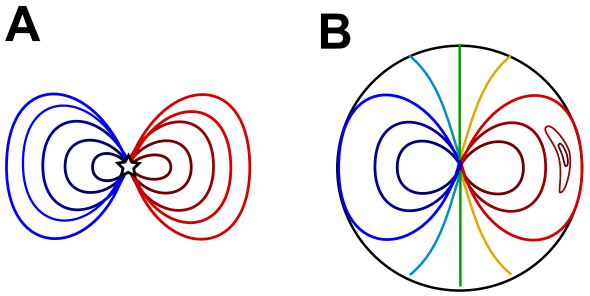

Figure S1: **Topologies.** The 8-shapes bouquet (A) and the topology of the dipole (B), with an isolated defect.

maximum and minimum values. Therefore we need to consider maps that have discontinuities. Simplest maps are those with one single singularity (e.g., at zero). We shall from now on consider continuous maps of the pointed disc ($\Omega^\times = \Omega \setminus \{0\}$). We aim at characterizing maps satisfying the assumptions:

(H1). *Regularity:* the map is smooth in the sense that all level sets are smooth curves.

(H2). *Exhaustivity:* the map is surjective in any neighborhood of 0;

(H3). *Parsimony:* the topological redundancy is minimal at any scale, i.e. there exist arbitrarily small topological discs around the singular point on which the representation achieves the minimal geometric redundancy.

The dipole is continuous (H1) and exhaustive (H2). We now prove that it is also parsimonious (H3).

Proposition I.2. *There is no continuous surjective map $g : \Omega^\times \mapsto \mathbb{R}$ with geometric redundancy 1.*

Proof : Let $u_0 \in g(\Gamma)$. If the level set of u_0 does not intersect the interior of Ω, then by continuity the values in the interior are either all larger or smaller than u_0 contradicting the surjectivity. Therefore, the level set enters the interior of Ω. If g has geometric redundancy one, $g^{-1}(u_0)$ is connected. If the singularity at the origin is not in its adherence, it cuts the disc in two parts, one of which containing the singularity. And by symmetry, we can assume that g is larger than u_0 on the part Ω' that does not contain the singular point (and smaller in the other domain). This set Ω' is compact, the map g reaches a finite maximum on that set, thus it cannot be surjective. The level set of u_0 therefore reaches the origin, and the complementary of the level set $\Omega \setminus g^{-1}(u_0)$ is connected. The values of the map, which is continuous on this set, do not intersect u_0, therefore are either larger or smaller than u_0, contradicting again the surjectivity. □

Therefore, minimal representations of the SF have at least geometric redundancy 2. In particular, the dipolar topology is parsimonious (H3). We now show that this dipolar architecture is actually the unique topology ensuring parsimony at small scales. This proof being slightly more involved, we state the result here, and provide the proof in section I.IV.

Theorem I.3. *Any map satisfying assumptions (H1), (H2) and (H3) at arbitrarily small scales around the singular point have the topology of the dipole.*

We conclude that, as is the case of the pinwheel topology for OR representations, not only the dipolar architecture is minimal in the sense of geometric redundancy, it is also the only one that satisfies this property: the dipolar architecture is universal.

I.IV Proof of the universality of the dipolar architecture

We call *8-shapes bouquet* an ensemble of level sets forming two lobes, each of which made of a collection of loops, connecting the singularity to itself. An 8-shape bouquet is represented in Fig S1A.

The level sets contained inside Ω are one of four types

1) simple closed curves,

2) arcs going from Γ to Γ,

3) arcs going from Γ to 0,

4) arcs with two extremities tending to 0.

It is clear that any continuous map g from the pointed disc $\Omega^\times$ to the real line $\mathbb{R}$ is continuous on Γ, and therefore levels connecting to the boundary necessarily represent a bounded set of values. The map hence necessarily displays level sets fully contained inside the disc Ω, i.e. that are either of type 1 or 4. Two types of level sets only add up to the redundancy: these are loops (type 1 level sets) which do not enclose the singularity and type 2 arcs. Indeed, if C is a closed level set that does not enclose the singularity, the level sets of g inside $I(C)$ only cover a bounded interval of values. Within any topological disc containing 0 and not enclosing C, exhaustivity and parsimony shall be satisfied, therefore one will find other connected components of all level sets represented within $I(C)$. In other words, C is an *isolate* in which all levels represented only add up unnecessary redundancy. Similarly, components of type 2 also create isolates (the domain enclosed between the curve of type 3 and Γ which does not contain the origin) which only add redundancy.

Besides this addition to the redundancy, the presence of such isolates may imply the presence of non-smooth level sets corresponding to the local maxima, contradicting the regularity assumption (H1). We therefore consider topologies with no such isolates, and will come back to this assumption at the end of the demonstration.

Therefore, the only components C of type 1 (i.e. closed curve) considered enclose the singularity in its interior $I(C)$ (which is well defined and homeomorphic to a disc by the Jordan and Schoenflies Theorem [1]). Within its interior, there cannot be only components of type 1: otherwise, in order to ensure exhaustivity, the map shall show major variations from very large to very low values, which is not possible with redundancy 2. The type 1 component therefore necessarily contains at least one component of type 4.

We now analyze the possible presence of these type 4 level sets (closed curves containing the singularity). If C is a component of type 4, by the Jordan-Schoenflies theorem, it encloses a domain Δ which is homeomorphic to a disc, and contains 0 in its boundary. It is easy to see that all level sets inside Δ are necessarily of type 4. On one component Δ, f may diverge to $+\infty$ or to $-\infty$, but cannot be doubly unbounded because of the parsimony assumption (shall the map have unbounded positive and negative values, it would present an infinite number of oscillations). These remarks allow now to demonstrate that any possible topology satisfying our assumptions contains a unique 8-shape bouquet.

Proposition I.4. *A map satisfying assumptions (H1), (H2) and (H3) contains a unique 8-shape bouquet, and values of g in each lobes are not overlapping.*

Proof: First of all, as we noted already, surjectivity of the map implies that the map necessarily has components at least two disconnected components of type 4. Indeed, otherwise all levels not represented on the boundary correspond to curves of type 1. The level sets within the interior of Ω is then made of nested closed loops circling the singularity, and it is not possible for the map to reach $-\infty$ and $+\infty$ with redundancy two. The map therefore necessarily has components of type 4. Within one component of type 4, the map may reach arbitrarily large (or arbitrarily small) values but not both. Let us assume that it reaches $+\infty$. We denote by Δ_+ the interior of the largest component of type 4 of this family. $\Omega \setminus \Delta_+$ has the topology of an annulus and shall represent all values until $-\infty$, and this is not possible with only arcs of type 1 by continuity. Indeed, let us for instance restrict the map to a topological circle Ω' containing the singularity and intersecting the boundary of Δ_+. Within Ω', components of type 1 are not possible because levels cannot cross the connected components of Δ. However, the map within $\Omega' \setminus \Delta_+$ represents all values within a semi-infinite interval $(-\infty, x_{\min}]$. It necessarily has level sets fully included within $\Omega' \setminus \Delta_+$, i.e. components of type 4.

By the redundancy 2 assumption, the map cannot have more than two lobes made of type 4 arcs. Indeed, at sufficiently small scale around the singularity, an arc of type 4 becomes two arcs of type 3, and by redundancy two assumption, this implies that there is no component reaching the singularity and corresponding to this level. In particular, different lobes cannot represent overlapping values (we would otherwise have 4 connected components for any common value at sufficiently small scale). The same argument may be applied to rule out the presence of strictly more than two lobes. Indeed, if this was the case, consider three levels $a < b < c$

belonging to different lobes. By continuity, there exists an additional connected component for the level set b disconnected from the type 4 arc considered in the lobe, and therefore redundancy 3 at small enough scale, which ends the proof. □

In order to complete the proof of Theorem I.3, we shall now characterize the possible topologies of level sets outside of the 8-shapes bouquet. Without loss of generality, we now consider a neighborhood of the singularity intersecting both lobes of the 8-shapes bouquet and in which the map satisfies (H1), (H2) and (H3). We denote by C_+ and C_- the external components of the two branches of the unique 8-shape bouquet converging to $\pm\infty$, c_+ and c_- the respective finite extremal values of f on C_+ and C_-. We have shown in the previous proof that necessarily $c_+ > c_-$.

The rest of the values are now shown to be represented along pairs of arcs of type 3.

Proposition I.5. *The map g is topologically equivalent[2] to a dipole. In particular, in addition to the 8-shapes bouquet, level sets are arcs of type 3 and the value of g goes monotonically from c_+ to c_- on both sides of the bouquet.*

Proof: The bouquet separates the rest of disc in two parts, said left (L) and right (R). On both parts, the map covers the whole interval (c_-, c_+), and therefore there is exactly one connected component of the level sets of g corresponding to the intervals (c_-, c_+) in L and in R. This connected component exists in arbitrarily small neighborhoods of the singularity. Since there is a single curve, it is connects the singularity to the boundary, and the redundancy 2 implies that f varies monotonically on them in L as well as in R. We therefore have pairs of arcs of type 3 for every level within (c_-, c_+) and arcs of type 4 in the interval $I_+ = (c_+, \infty)$ and $I_- = (-\infty, c_-)$. Let us show that there cannot be additional level sets (apart from small isolated defects). It is clear that additional level sets shall necessarily correspond to values in I_+ or I_- that are the only levels which, within Ω, have redundancy 1. By continuity, additional level sets therefore necessarily belong to the interior of the bouquet, and such arcs can therefore only be of type 1 and do not enclose the singularity: these are isolated defects. □

This concludes the proof of the theorem I.3: apart from isolated defects, the map has the topology of the dipole S1B. An example of the only possible type of defect is added in this picture.

II The angle distribution

In [2] it has been shown that the experimental distribution of the angle ψ between the level sets of OR and SF maps near the PC's (i.e., for distances smaller than $150\mu m$) can be roughly approximated with a uniform distribution, with a small significant bias towards small angles. We showed in the main text results that dipoles are good candidates to fit this distribution. We provided an analytically solvable model and simulations with other models, and claimed that essentially, distinct dipolar models can be precisely fitted to the experimental angles distribution.

Let us start by considering the maps $\varphi : re^{i\phi} \mapsto \frac{\phi}{2}$ for the OR and γ_α for the SF:

$$\gamma_\alpha : re^{i\phi} \mapsto \cos(\phi)/r^\alpha. \tag{S1}$$

It is straightforward to derive a formula for the intersection angle $\psi = h(\phi)$ between the iso-OR and iso-SF lines:

$$\cos\psi = \left| \frac{\sin(\phi)}{\sqrt{\alpha^2\cos^2(\phi) + \sin^2(\phi)}} \right|. \tag{S2}$$

Noticing that ϕ depends only on the angular coordinate ϕ, the corresponding probability density $P(\psi)$ in a circle around the pinwheel singularity, can be derived from the infinitesimal formula

$$d\phi = \frac{d\psi}{h'(h^{-1}(\psi))} \tag{S3}$$

[2]Mathematically, two maps $f : I(\Omega) \mapsto \mathbb{R}$ and $g : I(\Omega) \mapsto \mathbb{R}$ are said to be topologically equivalent if there exists an homeomorphism (smooth invertible map) φ of the open disc $I(\Omega)$ onto itself and a homeomorphism ψ of $\mathbb{R}$ onto itself such that: $f = \psi \circ g \circ \varphi^{-1}$.

and after normalisation reads

$$P(\psi) = \frac{2\alpha}{\pi\left((\alpha^2-1)\cos^2(\psi)+1\right)}. \tag{S4}$$

The case $\alpha = 1$ corresponds to a perfectly uniform distribution for the angles between iso-SF and iso-OR lines. The density $P(\psi)$ is a one-parameter function which can be fitted to the experimental data, and for any $\alpha < 1$, the qualitative experimental observation on the shape of the distribution is recovered. Optimizing on α using the best weighted least squares fit, we obtain an excellent fit with the experimental data shown in Fig. 1C. The best fitted distribution corresponds to $\alpha = 0.73$ (with 95% confidence bounds $(0.70; 0.76)$ and coefficient of determination 0.97).

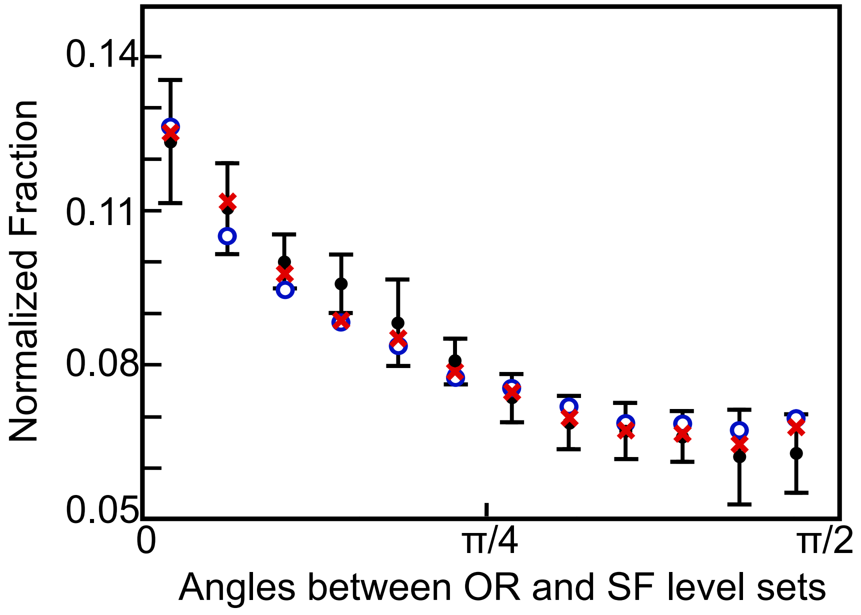

Figure S2: **Intersection angles distribution.** Distribution of the angles between the level sets of OR and SF maps locally around the pinwheels for different models. The black dots and the error bars represent respectively the means and standard deviations with respect to different cats from experimental data. Red crosses and blue circles corresponds respectively to the numerical results for the maps γ_B and γ_C defined in the SI Text (Eq. S5).

As we mentioned in the text, this is a systematic effect of saturation of the SF map close to the singularity, causing the deformation from a uniform distribution of angles to an excess in correspondence of small ψ.

In that sense, it is important to note that this observation is not strongly dependent on the model chosen. The interest of considering the previous γ_α case, is given by the fact that it allows for analytic developments. Other maps with saturating effects also reproduce qualitatively the experimental observation and allow very good fits with the experimental data. From the biological viewpoint, it is very natural to consider thresholded dipoles: on the one hand, it allows consistency with the data by avoiding a non-biological divergence of the SF map at the singularity, and on the other hand it takes into account the presence of the indeterminacy regions observed in the experimental data in the vicinity of the PCs [3], which shall not be in our model regions with a high number of level sets in order to avoid biasing the distributions. We consider here, as examples of such

maps, the one-parameter models:

$$
\begin{aligned}
\gamma_A &: re^{i\phi} \mapsto \max\left(-A, \min\left(A, \cos(\phi)\frac{R}{r}\right)\right) \\
\gamma_B &: re^{i\phi} \mapsto \cos(\phi)\tanh\left(\frac{B\,R}{r}\right) \\
\gamma_C &: re^{i\phi} \mapsto \cos(\phi)\ \mathrm{erf}\left(\frac{C\,R}{r}\right)
\end{aligned} \tag{S5}
$$

where R is the radius of the disc around a PC, and correct dimensions for SF are thought to be ensured by hidden units in front of each formula. Since in these cases ψ is a function of both coordinates r and ϕ, it is difficult to find an explicit formula for $P(\psi)$. However the numerical results (with $R = 50$ pixels), corresponding to the parameter choice $A = 1.4$, $B = 0.35$ and $C = 0.3$, and represented in Fig. 1C (γ_A) and in Fig. S2 (γ_B and γ_C), all show an excellent fit with the experimental data. Therefore, a large family of dipolar architectures can quantitatively reproduce the experimental data. Our experimental techniques do not allow to discriminate between them at the level of the angle distribution; for our purposes, as shown in the next section, the fine structure of the map can be taken into account in an effective way by suitably fixing the parameters of a chosen model.

III Neural Coding Model

In order to test coding capabilities of the SF-OR representation architectures, we have defined a model of OR-SF map based on our theory, and fitted to our experimental observations. We describe in this section the features of the model.

III.I Geometrical descriptions of the OR and SF maps

We consider a circular region (the pinwheel area, PA) around a PC, and use polar coordinates for the parametrization of the points. For simulations the PA was embedded into a disc of radius $R = 50$ pixels. Two structures have been considered for the SF map: the dipolar architecture observed experimentally in [3], and a putative orthogonal architecture with a local SF extremum located at the PC [5, 6, 7] consistent with the uniform coverage principle.

III.I The orientation map:

Classical neuro-geometrical theory [8] describes the OR map in the PA as the half angle of the coordinate, defined modulo π:

$$\theta(r, \phi) = \phi/2 + \phi_0, \tag{S6}$$

where ϕ_0 is a phase arbitrarily chosen and different for every pinwheel.

III.II The dipolar SF map:

From the mathematical viewpoint, an idealised map displaying dipolar architecture in the PA is given by the family of functions γ_α defined in Eq. S1, with $\alpha > 0$. This α-dependent functions exhibits a singularity at the PC (r=0) where it reaches both its minimal and maximal value and shows circular level sets 180° apart. In order to catch the variability in the angular coordinates, for the numerical studies we allowed moreover deformations of this idealised model, without changing the topology of the level sets. The SF map structure in the PA was then defined as follows:

$$\nu_{dip}(r, \phi) = \mathbb{H}_{\nu_{\min}}^{\nu_{\max}}\left(\mu\left(\frac{R}{r\sqrt{\cos^2(\phi)\cos^2(\chi) + \sin^2(\phi)\sin^2(\chi)}}\right)^{\alpha}\cos(\tilde{\phi}) + \nu_0\right), \tag{S7}$$

where $\mathbb{H}_{\nu_{\min}}^{\nu_{\max}}$ is the identity map saturating below at $\nu_{\min}$ and above at $\nu_{\max}$, and $\tilde{\phi} = \phi + 2\pi\zeta\cos(\phi)$, is an angular deformation allowing architectures in which circular level sets are not 180° apart. The parameters α, μ, χ, ζ and ν_0 allow continuous deformations of the shape of the iso-SF lines. As discussed in the main text and in the previous section, thresholds at a maximum and minimum SF values ν_{max} and ν_{min} were added in Eq. S7. In order to evaluate the most reliable values for these parameters to be used for the numerical studies, the model was fitted via a least-square method to the optical imaging data. We first focused on the angular variability, neglecting the dependence on α and fixing the simplest case $\alpha = 1$. Only fits with coefficient of determination > 0.8 (i.e. fits for which 80% in the response variable can be explained) were kept, analogously to what has been done in [3]. Matlab function *regress* was used to this purpose. In a second step, we studied the dependence of our results from the density of level sets in the radial direction, by choosing other values for α and adjusting the other parameters to suitably fit the data.

The mean value of preferred SF on the global map increases from anterior to posterior region of the cortex [9]; however for our purposes, we are mainly interested in the local relative values, and in particular on the difference $\Delta\nu_{extr} = \nu_{max} - \nu_{min}$. The data show that $\Delta\nu_{extr}$ depends on the area of SF indeterminacy cited above. This is consistent with the idealized dipole model that locates extrema at the singularity: the smaller the indeterminacy area, the larger the SF range. The best value after linear regression (mean $\pm$ standard deviation) is given by $\Delta\nu_{extr} = 2.3 \pm 0.8$ octaves [3]. Moreover, the data show that (i) μ can be well approximated as linearly dependent on the difference between the local extreme SF values, and (ii) ν_0 by their average. We consider then $\mu = \mu'\Delta\nu_{extr}/2$ and $\nu_0 = (\nu_{max} + \nu_{min})/2 + \nu_0'$, and all in all we can rewrite

$$\nu_{dip}(r,\phi) = \max\left(-\frac{\Delta\nu_{extr}}{2}, \min\left(\frac{\Delta\nu_{extr}}{2}, \tilde{\nu}_{dip}(r,\phi)\right)\right),$$

$$\tilde{\nu}_{dip}(r,\phi) = \mu'\frac{\Delta\nu_{extr}}{2}\left(\frac{R}{r\sqrt{\cos^2(\phi)\cos^2(\chi) + \sin^2(\phi)\sin^2(\chi)}}\right)^{\alpha}\cos(\phi + 2\pi\zeta\cos\phi) + \nu_0' \qquad \text{(S8)}$$

The parameters $\alpha, \mu', \chi, \zeta, \nu_0'$ obtained by the fits and successively used in the simulations are listed in Table A.

Table A: **Parameters of the model fitted on experimental data.**

α	0.5	0.75	1	1.25	1.5
μ'	0.7 ± 0.3	0.5 ± 0.2	0.4 ± 0.2	0.4 ± 0.2	0.3 ± 0.2
χ	0.4 ± 0.5	0.6 ± 0.5	0.7 ± 0.4	0.7 ± 0.3	0.7 ± 0.3
ζ	0.0 ± 0.3	0.0 ± 0.3	0.0 ± 0.3	0.0 ± 0.3	0.0 ± 0.3
$\nu_0'^{\dagger}$	0.0 ± 0.5	0.0 ± 0.4	0.0 ± 0.4	0.0 ± 0.4	0.0 ± 0.3

† Expressed in octaves.

III.III The orthogonal SF map:

Orthogonal architectures in the PAs were not observed in our experimental data. The model we chose is based on the principle of uniform coverage that was argued as an advantageous representation of OR and SF. The ideal orthogonal architecture ensuring an optimal uniform coverage is given by a linear dependence of the SF level sets as a function of the radius r. Such orthogonal architectures present one extremum at the singularity. The perfect orthogonal pinwheel presenting a maximum of the SF at the singularity is given by the map:

$$\nu_{pol}(r,\phi) = \nu_{max} - \beta\, r. \qquad \text{(S9)}$$

The parameter β was extrapolated from the experimental data. In particular, considering a pair of pinwheels at a distance d on the cortex and representing a maximum and a minimum (Fig. S3A), the slope is identified as $\beta = \Delta\nu_{extr}/d$. We therefore estimated d as the average minimal distance between PCs observed in our

optical imaging data, and in particular it reads $d = 450 \pm 90\mu m$ (Fig. S3B). Again, we can consistently take $\nu_{max} = \Delta\nu_{extr}/2$.

Since this architecture is purely theoretical, we do not consider further deformations for the Eq. S9: notice that in general, adding a dependence of $\nu_{pol}(r, \phi)$ on the angle ϕ would worst the precision of the detection for this kind of architecture. Changing ν_{max} into ν_{min} and β into $-\beta$ would provide an architecture with a minimum at the PC.

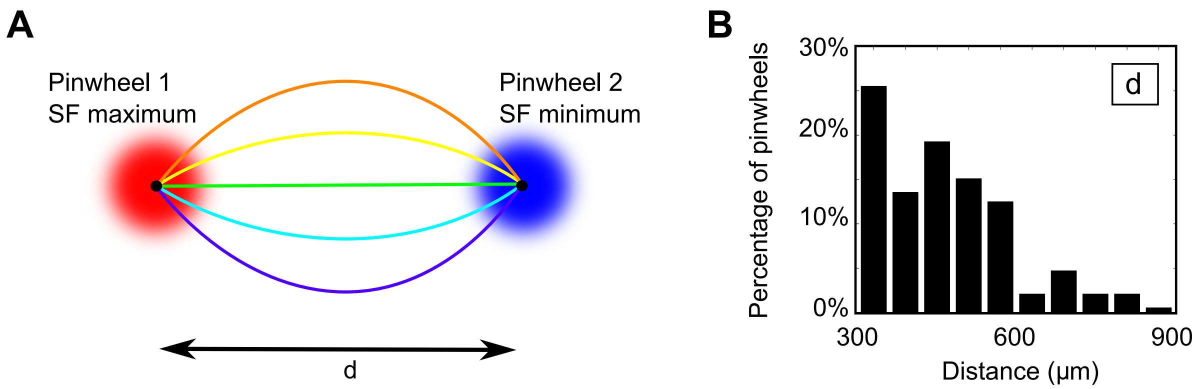

Figure S3: **Estimation of the parameters for the orthogonal architecture.** (A): Schematic representation of the model used for the orthogonal architecture (adapted from [7]). In this model, iso-OR lines (colored) intersect at PCs that tend to lie over low (red) and high (blue) SF domains. In order to represent all combinations of OR and SF equally, SF was assumed to linearly vary between the two pinwheels separated by a distance d. The value of d was defined as the median minimum distance (± mad) from one pinwheel to another whose histogram is shown in (B).

Under these assumptions, the role of functional map geometry is to determine which part of the parameter space is locally represented in a given cortical area, as shown in Fig. 2A and Fig. 2B. Notice in particular that, since the dipolar architecture is not rotationally invariant (in contrast to the orthogonal), the phase ϕ_0 in Eq. S6 would reflect in a different coverage of the parameter space (compare for instance Fig. 2A, corresponding to $\phi_0 = 0$, and Fig. 6D, where $\phi_0 = \pi/2$).

III.II Tuning Curves

A pixel with preferred OR-SF (θ^*, ν^*) elicits a response with amplitude $F_{\theta^*\nu^*}(\theta_{st}, \nu_{st})$ to a stimulus with OR and SF attributes (θ_{st}, ν_{st}) that achieves its maximum at (θ^*, ν^*). For simplicity the tuning curve $F_{\theta^*\nu^*}(\theta, \nu)$ was approximated as the product of two functions $F^1_{\theta^*}$ and $F^2_{\nu^*}$

$$F_{\theta^*\nu^*}(\theta, \nu) = F^1_{\theta^*}(\theta)\, F^2_{\nu^*}(\nu) \tag{S10}$$

where $F^1_{\theta^*}$ (resp. $F^2_{\nu^*}$) is the tuning curve for OR (resp. SF) detection. The OR tuning curve $F^1_{\theta^*}$ was modeled as a wrapped Gaussian function [10]:

$$F^1_{\theta^*}(\theta) = \mathcal{N}\, \Sigma^N_{n=-N} e^{-\frac{(\theta-\theta^*+n\pi)^2}{2\sigma^2_{OR}}}, \tag{S11}$$

where σ_{OR} is a measure of the width of the tuning curve and $\mathcal{N}$ is the normalisation factor. $N = 3$ is sufficient to accurately describe the OR tuning response in the experimental data [11].

The SF tuning curve $F^2_{\nu^*}$ is modeled as a Gaussian function with a standard deviation σ_{SF}:

$$F^2_{\nu^*}(\nu) = \frac{1}{\sqrt{2\pi}\sigma_{SF}} e^{-\frac{(\nu-\nu^*)^2}{2\sigma^2_{SF}}}. \tag{S12}$$

The Full Width at Half Height (FWHH) of the SF tuning width corresponds to $w = 2\sqrt{2\log 2}\,\sigma_{SF}$. The tuning width plays an important role in the coverage of the parameter space and on the coding capabilities, as we discussed in the main text. The sharper the tuning curve, the closer to Fig. 2 of the main text the response to pairs (OR, SF) will be, and the flatter the SF tuning curve, the more uniform the responses will be. Fig. S4 show the level of response of a specific architecture to a pair (OR, SF) in the map corresponding to Fig. 2. The histogram of response amplitude appears on the upper right corner. We see that in the dipolar architecture, very few pixels elicit small responses, while in the orthogonal architecture, a substantial part of the (OR, SF) plane does not elicit any response. This can probably help efficient coding and decoding.

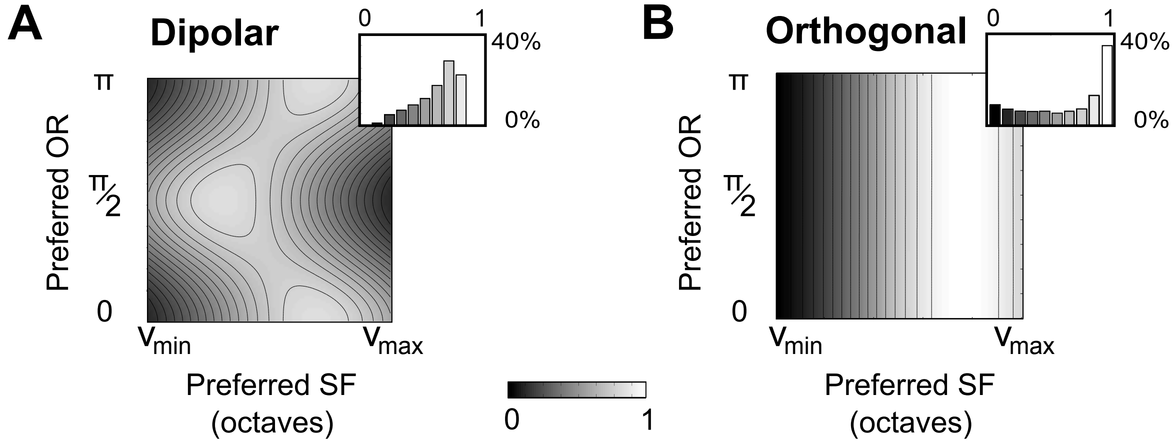

Figure S4: **Representation of the induced activity in the parameter space.** Relative response of dipolar (A) and orthogonal (B) architectures near PCs to external stimuli in the parameter space. For any given pixel (θ^*, ν^*), we show the normalised sum of all the responses $F_{\theta^*\nu^*}(\theta_{st}, \nu_{st})$ (Eq. S10, S11 and S12, with parameters $\sigma_{OR} = 0.63$ and $\sigma_{SF} = 1$ octaves) with stimuli (θ_{st}, ν_{st}) spanning the same parameter space. The normalisation is chosen by taking the minimum and maximum values of the two combined distributions. The relative distributions are represented in the histograms in the top-right corners.

III.III The coding and decoding efficacy of dipolar and orthogonal architectures

Once the architecture and selectivity properties are defined, we dispose of the set of responses $F_{\theta_i^*\nu_i^*}(\theta_{st}, \nu_{st})$ to a stimulus (θ_{st}, ν_{st}), for all the different pixels i in the cortical region under consideration. We use this information:

- to obtain an estimation of the capability of both two architectures to discriminate between two similar gratings, and
- to obtain an estimation of the error made in evaluating the stimulus that elicited this response pattern in a PA.

Discriminating similar visual stimuli We drawn a set of 200 different dipolar maps (Eq. S8) and 200 orthogonal maps (Eq. S9) according to the fitted distribution of parameters. We presented to each architecture 100 random stimuli (θ_{st}, ν_{st}) drawn uniformly in the square $[0, \pi) \times [\nu_{min}, \nu_{max}]$, with $\nu_{max} = -\nu_{min} = \Delta\nu_{extr}/2$. We fixed the FWHH of the OR tuning curves to the typical value reported in the literature near PC $y_{exp} = 80°$ [12]. As for the SF FWHH, we used two benchmark points $w = 2.48$ octaves (value reported at the whole map scale [2]) and $w = 1.83$ octaves (value found near PCs in the present study, see Fig. 6).

For each stimulus (θ_{st}, ν_{st}), we chose a stimulus at a distance δ: $(\theta_{st} + \delta\theta, \nu_{st} + \delta\nu)$ with $\delta\theta = \delta \cdot \cos(\eta) \cdot \pi$, $\delta\nu = \delta \cdot \sin(\eta) \cdot \Delta\nu_{extr}$ and ν uniformly distributed in $[0, 2\pi]$. We considered four different values for the distance

$\delta = 5 \cdot 10^{-3}, 10^{-2}, 5 \cdot 10^{-2}, 10^{-1}$. For each couple of stimuli and a given map, we evaluated the norm of the difference in the cortical response in the PA, namely

$$\|\Delta A\|_2 = \sqrt{\sum_{i \in \mathrm{PA}} \left(F_{\theta_i^* \nu_i^*}(x) - F_{\theta_i^* \nu_i^*}(x')\right)^2} \tag{S13}$$

with $x = (\theta_{st}, \nu_{st})$ and $x' = (\theta_{st} + \delta\theta, \nu_{st} + \delta\nu)$ and i runs over the set of pixels in the PA. For two nearby stimuli, most pixels will display small errors washed out by noise. In order to prevent amplification of these non-informative events, we only considered pixels at amplitude larger than 1% of the maximal activity in the PA ($F_{\theta_i^* \nu_i^*}$). Note that very similar results occur for non thresholded $\|\Delta A\|_2$. We then compared the distribution of $\|\Delta A\|_2$ across the different dipolar and orthogonal maps in two different ways. First we obtained, for each pinwheel, a median value by varying the stimuli and compared the medians and m.a.d. over this distribution, as a function of δ. As shown in the main text in Fig.4B, we obtained two almost perfectly linear curves. In particular the slope of the curve relative to the dipolar architectures is larger than the orthogonal one. As a result, if the actual discrimination between two similar gratings is indeed more efficient for larger $\|\Delta A\|_2$, the dipolar architecture would allow a finer discrimination than the orthogonal architecture. This can also be shown by comparing the distributions of the difference of medians, varying the stimuli, of randomly chosen couple of dipolar and orthogonal structures. This is shown in in Fig.4C.

Decoding and errors In order to extract a unique value for the estimations (θ_{ev}, ν_{ev}) of the external stimulus, we took the average value of (θ_j^*, ν_j^*) within the 10% highest amplitudes, weighted by the response amplitude $F_{\theta_j^* \nu_j^*}(\theta_{st}, \nu_{st})$. The actual mechanism used by the cortex is probably more involved, but this strategy can be seen as a reasonable first approximation of the process, or alternatively as a breech towards understanding the role of architecture in coding capabilities[3].

In this case, we considered a set of $N_{PC} = 50$ different dipolar maps and $N_{PC} = 50$ orthogonal. Again, we tested for 100 random stimuli and we first fixed the FWHH of the OR and SF tuning curves $y_{exp} = 80°$ and $w = 2.48$ octaves, in order to compare the errors for dipolar and uniform coverage architectures. The errors ϵ_θ and ϵ_ν are defined as:

$$\begin{cases} \epsilon_\theta = |\theta_{ev} - \theta_{st}| / \pi, \\ \epsilon_\nu = |\nu_{ev} - \nu_{st}| / \Delta\nu_{extr}. \end{cases} \tag{S14}$$

Moreover, we repeated the same analysis for other values of the SF tuning width (see Fig. 5 of the main text).

The distribution of the normalized errors is highly asymmetric. For such distributions, median and median average deviation (mad) are the relevant statistical measures. Again, we obtain, for each pinwheel, a median error. This collection of 50 medians allows computing the typical errors $(\epsilon_\theta, \epsilon_\nu, \epsilon_{tot})$ as the median of these distributions, as well as the deviations around these values (mad).

Statistics related to differences between normalized errors were calculated using a two-sided Mann-Whitney-Wilcoxon test in Matlab *ranksum* function. The sample size was calculated with G*Power [13], given a power equal to 0.8, an effect size of 0.8 and a probability of rejecting the null hypothesis of 0.001.

III.I Estimating the intersection values between the error curves

The intersection point between ϵ_θ and ϵ_ν for the dipolar architecture was evaluated by fitting the error data with straight lines. To this end, we increased the number of different dipolar and orthogonal maps simulated ($N_{PC} = 200$) and used a weighted least-squares error method for the distributions of medians described above (weights are related to the dispersion of the data).

[3] Of course more complicated strategies for perception could be designed, which potentially depend on functional map topology. Designing in a relevant manner such procedure is probably premature due to the current limited understanding of perception mechanisms at the cellular level. The choice of the present coding-decoding procedure is one of the simplest assumption, and provide insight on how topology affects coding and decoding.

Table B: **Fits with affine functions**

α	0.5	0.75	1	1.25	1.5
a_θ	-1.8 ± 0.4	-1.6 ± 0.4	-1.4 ± 0.4	-1.5 ± 0.4	-1.4 ± 0.4
b_θ	9.6 ± 1.2	8.0 ± 1.2	7.3 ± 1.2	7.6 ± 1.1	7.0 ± 1.1
$\|r_\theta\|$	0.98	0.98	0.97	0.98	0.98
a_ν	5.5 ± 0.4	5.2 ± 0.3	4.7 ± 0.3	4.2 ± 0.3	4.1 ± 0.3
b_ν	-3.7 ± 0.6	-3.8 ± 0.6	-3.7 ± 0.5	-3.0 ± 0.4	-3.0 ± 0.4
$\|r_\nu\|$	0.99	0.97	0.99	0.98	0.98
$\overline{w}$	1.83 ± 0.23	1.74 ± 0.24	1.79 ± 0.25	1.88 ± 0.26	1.85 ± 0.27
ϵ	0.06 ± 0.01	0.05 ± 0.01	0.05 ± 0.01	0.05 ± 0.01	0.04 ± 0.01
Z-score	0.01	0.24	0.11	0.14	0.05
p-value	0.99	0.81	0.91	0.89	0.96

Intersections of ϵ_θ and ϵ_ν curves for different values of α, as defined in Sec. III.I. The coefficients a_θ and a_ν have the dimension of inverse octaves. The Z-scores and the two-sided p-values correspond to the null-hypothesis $\overline{w} = w_{exp}^{PC17}$, where $\overline{w}$ (expressed in octaves) is the value of the FWHH for SF at the trade-off, and $w_{exp}^{PC17} = 1.83 \pm 0.20$ octaves (median ± mad) is the experimental result within 25 μm from PCs in A17.

The linear fits for the error curves, corresponding to variations of the FWHH for the SF, named here w, are given by:

$$\epsilon_\theta = 10^{-2}\,(a_\theta\ w + b_\theta)\,,$$
$$\epsilon_\nu = 10^{-2}\,(a_\nu\ w + b_\nu)\,, \tag{S15}$$

for $1 \leq w \leq 4$ octaves and $a_\theta, b_\theta, a_\nu$ and b_ν listed in Table B, together with the correlation coefficients $|r_\theta|$ and $|r_\nu|$. The coefficients in front of w have the dimension of inverse octaves. The values $\overline{w}$ of the tuning width at the intersection are also listed in Table B for different choice of the parameter α, together with the relative errors for $\epsilon = \epsilon_\theta = \epsilon_\nu$, and are represented in the Fig. 7. In particular, we show, in terms of Z-scores and corresponding p-values, that the theoretical trade-off is compatible with the experimental results $w_{exp}^{PC17} = 1.83 \pm 0.20$ octaves, when the analysis is restricted within 25 μm from PCs in A17 [4]. It is also evident that the tuning width values obtained by the balance detection argument are not compatible with the value $w_{exp}^{all} = 2.48 \pm 0.19$ octaves experimentally obtained by considering the whole V1 surface [2] (as discussed in the main text).

The experimental values for the SF tuning width w are obtained in terms of median and mad. In order to evaluate the Z-scores, the robust estimate of the standard deviation (s.d.) was calculated with the formula: s.d. = 1.4826 mad. The difference of the values between the experimental data and the balanced detection point provide a value to be compared with zero, and the s.d. of the intersection point is obtained by error propagation method (valid since experimental and simulated data are statistically independent). From these data, we use the Z-score in two ways: (i) in order to ascertain whether two quantities are significantly distinct, we use the one-sided test, and (ii) the two-sided test in order to confirm if the two values are compatible.

Finally notice that a correspondent trade-off can be obtained for the dipolar architecture also by varying the FWHH for OR. For example, if the SF width is fixed to $w = 2.48$ octaves, the intersection of the two ϵ_ν and ϵ_θ curves points for OR towards larger FWHH than the standard 80°. Once again no balance detection is observed for the orthogonal architecture.

References

[1] Moise EE. Geometric topology in dimensions 2 and 3. Springer; 1977.

[4] Similar results are obtained when compared to the experimental value $w_{exp}^{PC18} = 1.83 \pm 0.24$ octaves obtained in A18.

[2] Ribot J, Aushana Y, Bui-Quoc E, Milleret C. Organization and origin of spatial frequency maps in cat visual cortex. The Journal of Neuroscience. 2013;33(33):13326–13343.

[3] Ribot J, Romagnoni A, Milleret C, Bennequin D, Touboul J. Pinwheel-dipole configuration in cat early visual cortex. bioRxiv doi: 101101/009308. 2014;.

[4] Kalatsky VA, Stryker MP. New paradigm for optical imaging: temporally encoded maps of intrinsic signal. Neuron. 2003;38(4):529–545.

[5] Shoham D, Hübener M, Schulze S, Grinvald A, Bonhoeffer T. Spatio–temporal frequency domains and their relation to cytochrome oxidase staining in cat visual cortex. Nature. 1997;385(6616):529–533.

[6] Issa NP, Trepel C, Stryker MP. Spatial frequency maps in cat visual cortex. The Journal of Neuroscience. 2000;20(22):8504–8514.

[7] Issa NP, Rosenberg A, Husson TR. Models and measurements of functional maps in V1. Journal of neurophysiology. 2008;99(6):2745–2754.

[8] Petitot J. Neurogéométrie de la vision: modèles mathématiques et physiques des architectures fonctionnelles. Editions Ecole Polytechnique; 2008.

[9] Tani T, Ribot J, O'Hashi K, Tanaka S. Parallel development of orientation maps and spatial frequency selectivity in cat visual cortex. European Journal of Neuroscience. 2012;35(1):44–55.

[10] Batschelet E. Circular statistics in biology. vol. 371. Academic Press London; 1981.

[11] Swindale NV. Orientation tuning curves: empirical description and estimation of parameters. Biological cybernetics. 1998;78(1):45–56.

[12] Rao SC, Toth LJ, Sur M. Optically Imaged Maps of Orientation Preference in Primary Visual Cortex of Cats and Ferrets. Journal of Comparative Neurology. 1997;387(3):358–370.

[13] Faul F, Erdfelder E, Buchner A, Lang AG. Statistical power analyses using G*Power 3.1: Tests for correlation and regression analyses. Behavior Research Methods. 2009; 41:1149–1160.
