# Supplementary figures and images for: Parsimony, Exhaustivity and Balanced Detection in Neocortex
Source: PLoS Comput Biol. 2015 Nov 20;11(11):e1004623. doi: 10.1371/journal.pcbi.1004623 (PMC4654526; doi:10.1371/journal.pcbi.1004623)

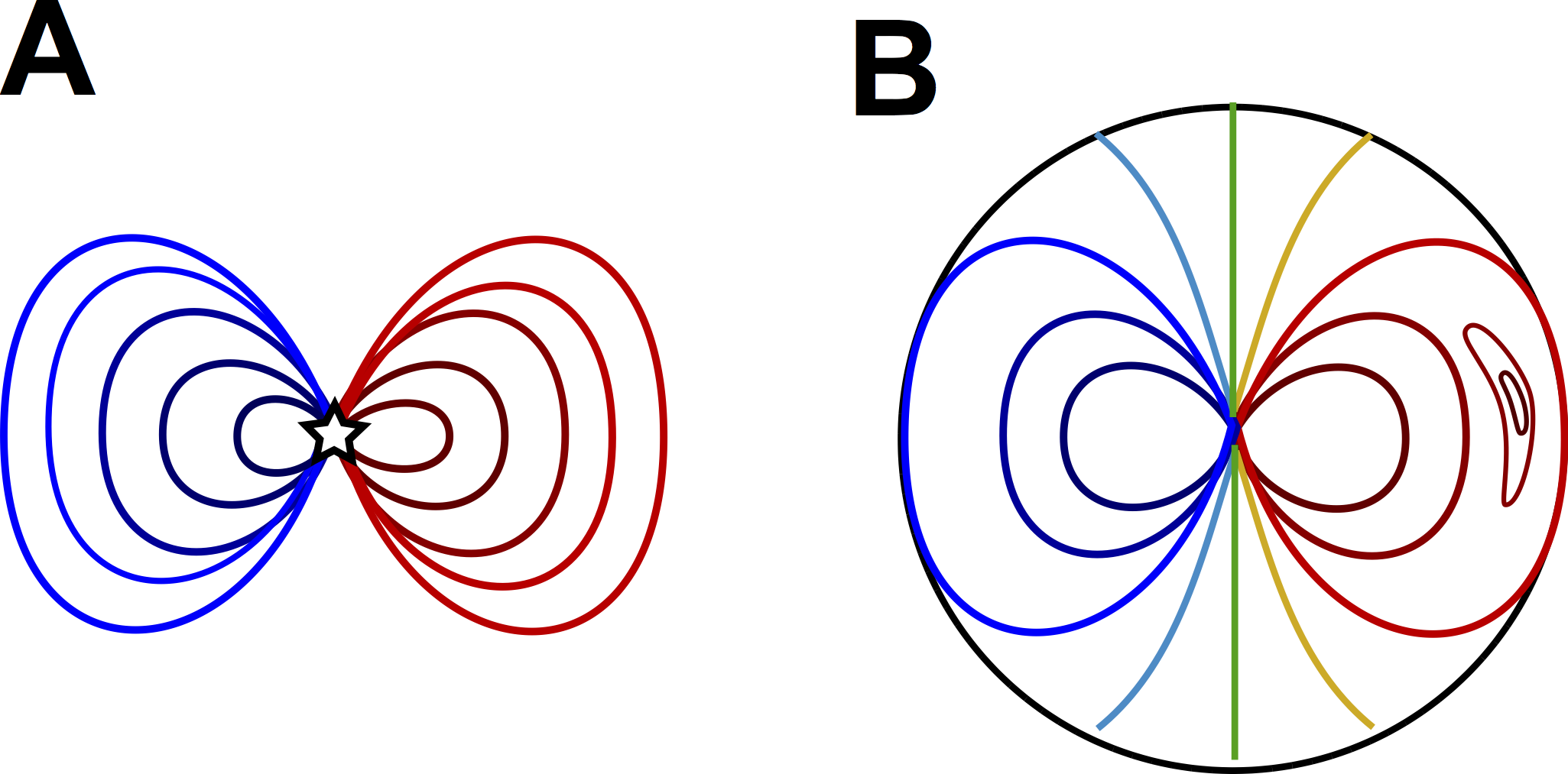

Supplement: S1 Fig — The 8-shapes bouquet (A) and the topology of the dipole (B), with an isolated defect. (TIFF) [file pcbi.1004623.s002.tiff]

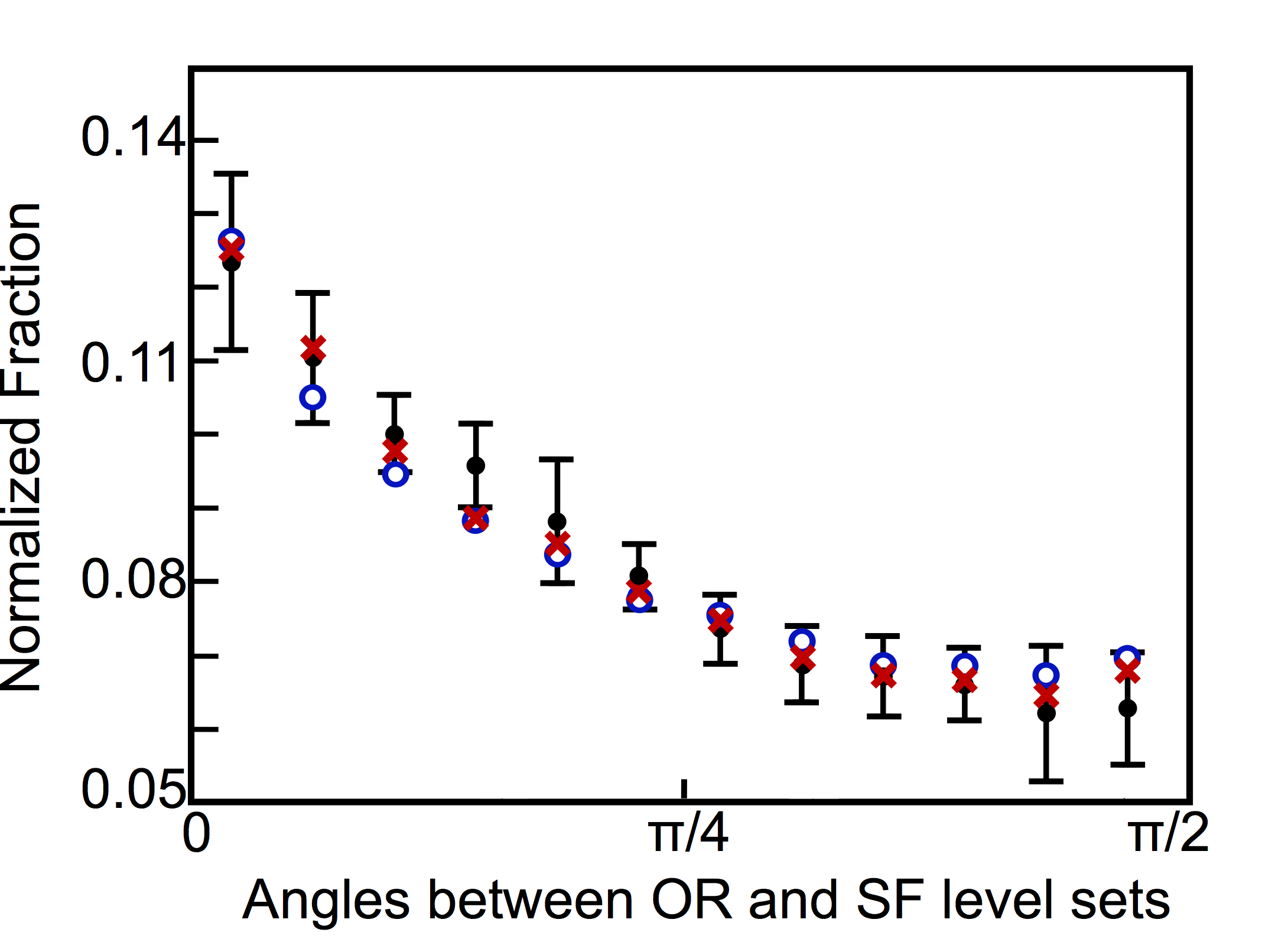

Supplement: S2 Fig — Distribution of the angles between the level sets of orientation and spatial frequency maps locally around the pinwheels for different models. The black dots and the error bars represent respectively the means and standard deviations with respect to different cats from experimental data. Red crosses and blue circles corresponds respectively to the numerical results for the maps γ B and γ C defined in the S1 Text. (TIFF) [file pcbi.1004623.s003.tiff]

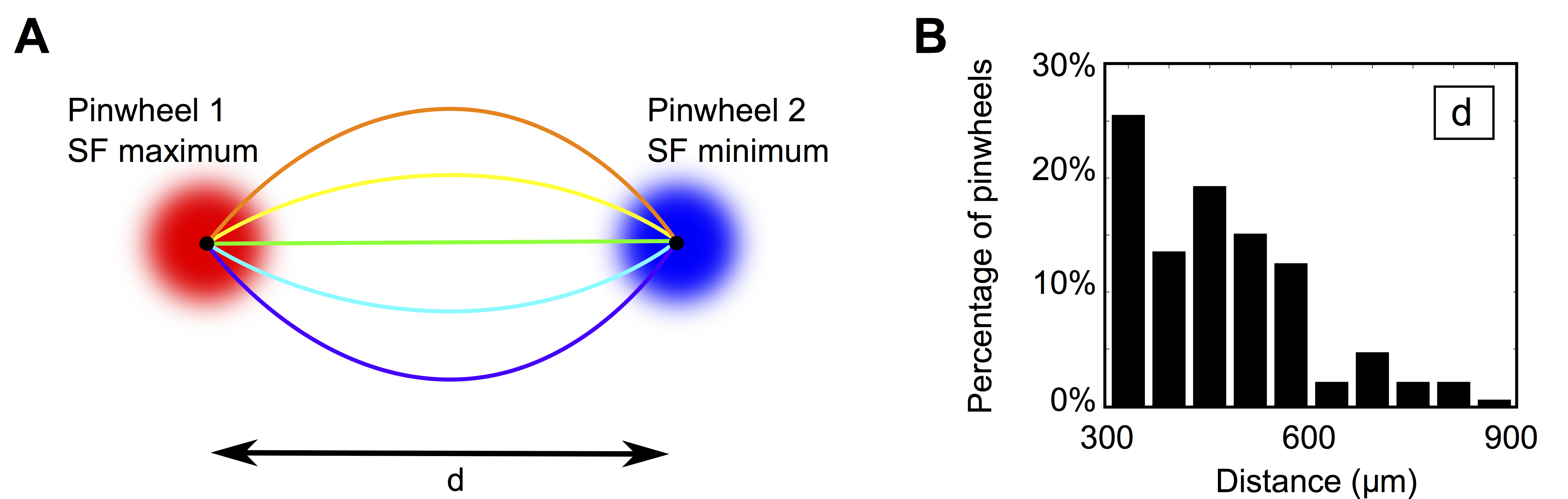

Supplement: S3 Fig — (A): Schematic representation of the model used for the orthogonal architecture (adapted from [15]). In this model, iso-orientation lines (colored) intersect at pinwheel centers that tend to lie over low (red) and high (blue) SF domains. In order to represent all combinations of OR and SF equally, SF was assumed to linearly vary between the two pinwheels separated by a distance d. The value of d was defined as the median minimum distance (± mad) from one pinwheel to another whose histogram is shown in (B). (TIFF) [file pcbi.1004623.s004.tiff]

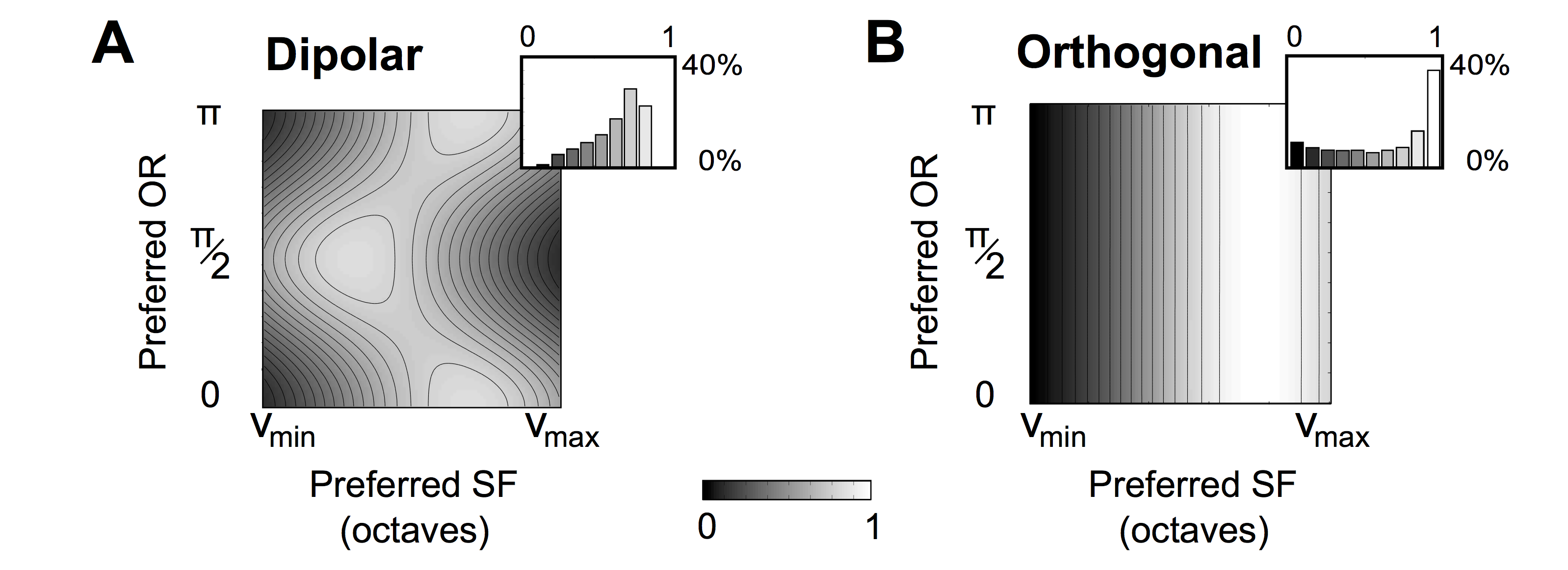

Supplement: S4 Fig — Relative response of dipolar (A) and orthogonal (B) architectures near pinwheel centers to external stimuli in the parameter space. For any given pixel (θ*, ν*), we show the normalised sum of all the responses F θ* ν*(θ st, ν st) (Eq. S10, S11 and S12 in S1 Text, with parameters σ OR = 0.63 and σ SF = 1 octaves) with stimuli (θ st, ν st) spanning the same parameter space. The normalisation is chosen by taking the minimum and maximum values of the two combined distributions. The relative distributions are represented in the histograms in the top-right corners. (TIFF) [file pcbi.1004623.s005.tiff]

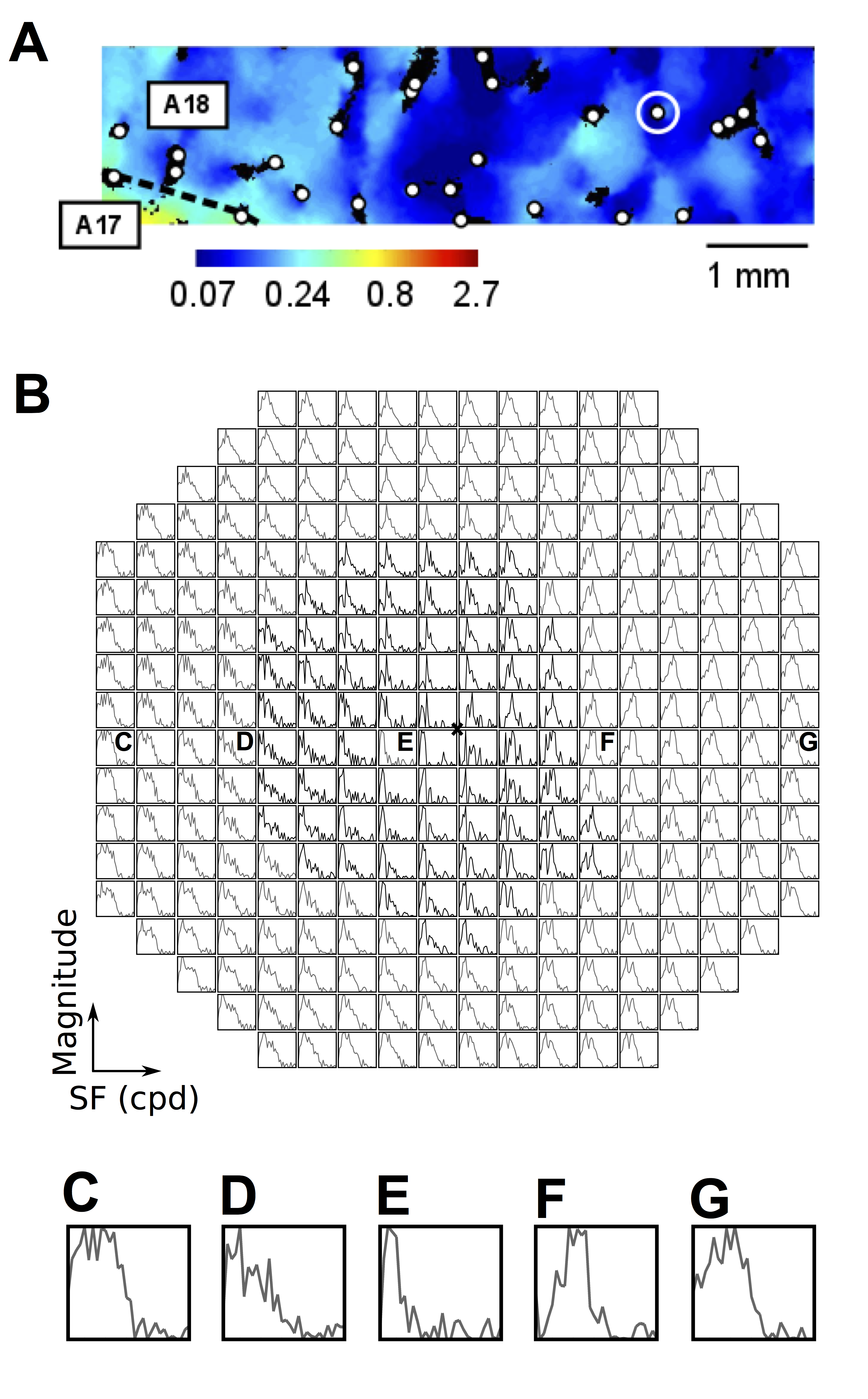

Supplement: S5 Fig — SF tuning curve relative to cortical area in the white circle in A are represented in B. Black curves represent pixels with a high error-of-fit (> 0.5). These are represented in the close vicinity of the PC. Gray curves represent pixels with an acceptable error-of-fit (< 0.5). Although most of these pixels are located further away (See C, D, F, G for instance), some are also located close to the PC (E). Surprisingly, the later pixels exhibit a sharp selectivity for SF preference. (TIFF) [file pcbi.1004623.s006.tiff]
